# A multidomain connector links the outer membrane and cell wall in phylogenetically deep-branching bacteria

**DOI:** 10.1073/pnas.2203156119

**Published:** 2022-08-09

**Authors:** Andriko von Kügelgen, Sofie van Dorst, Vikram Alva, Tanmay A. M. Bharat

**Affiliations:** ^a^Structural Studies Division, MRC Laboratory of Molecular Biology, Cambridge CB2 0QH, United Kingdom;; ^b^Sir William Dunn School of Pathology, University of Oxford, Oxford OX1 3RE, United Kingdom;; ^c^Department of Protein Evolution, Max Planck Institute for Biology Tübingen, Tübingen 72076, Germany

**Keywords:** outer membrane protein, cryo-EM, *Deinococcus radiodurans*, bioinformatics, bacterial cell surface

## Abstract

*Deinococcus radiodurans* is a phylogenetically deep-branching extremophilic bacterium that is remarkably tolerant to numerous environmental stresses, including large doses of ultraviolet (UV) radiation and extreme temperatures. It can even survive in outer space for several years. This endurance of *D. radiodurans* has been partly ascribed to its atypical cell envelope comprising an inner membrane, a large periplasmic space with a thick peptidoglycan (PG) layer, and an outer membrane (OM) covered by a surface layer (S-layer). Despite intense research, molecular principles governing envelope organization and OM stabilization are unclear in *D. radiodurans* and related bacteria. Here, we report a electron cryomicroscopy (cryo-EM) structure of the abundant *D. radiodurans* OM protein SlpA, showing how its C-terminal segment forms homotrimers of 30-stranded β-barrels in the OM, whereas its N-terminal segment forms long, homotrimeric coiled coils linking the OM to the PG layer via S-layer homology (SLH) domains. Furthermore, using protein structure prediction and sequence-based bioinformatic analysis, we show that SlpA-like putative OM–PG connector proteins are widespread in phylogenetically deep-branching Gram-negative bacteria. Finally, combining our atomic structures with fluorescence and electron microscopy of cell envelopes of wild-type and mutant bacterial strains, we report a model for the cell surface of *D. radiodurans*. Our results will have important implications for understanding the cell surface organization and hyperstability of *D. radiodurans* and related bacteria and the evolutionary transition between Gram-negative and Gram-positive bacteria.

## Significance

*Deinococcus radiodurans* is an extremophilic bacterium that has been studied intensely due to its hyperstability and deep position in the evolutionary tree of life, respectively. An atypical cell envelope is one factor underlying its hyperstability; however, molecular organizational principles central to this envelope remain unclear. We have solved the atomic structure of a highly abundant protein, SlpA, and discovered that it forms extended structures that link the outer membrane (OM) to the peptidoglycan (PG). SlpA-like putative OM–PG connector proteins are widely present in many Gram-negative phyla, where they likely play a key role in organizing the bacterial cell envelope. Our results will have important implications for understanding the organization and evolution of bacterial cell surfaces.

*Deinococcus radiodurans* is an evolutionarily deep-branching bacterium with several distinctive characteristics (1). It is remarkably tolerant to large doses of ionizing and ultraviolet (UV) radiation, extreme temperatures, osmotic pressure, oxidative stress, and desiccation, primarily owing to its extensive DNA repair system (2), complex cell envelope (3), and antioxidation systems, such as the one involving the carotenoid deinoxanthin (4, 5). In fact, *D. radiodurans* can even survive for years in outer space (6). Due to its ability to survive under extreme environmental conditions and its deep position in the bacterial tree of life, *D. radiodurans* has been of tremendous interest for several synthetic biology and evolutionary studies (2).

The cell envelope of *D. radiodurans* is atypical. While it stains Gram positive, its architecture resembles that of Gram-negative bacteria, containing an inner membrane (IM) covered by a peptidoglycan (PG) layer in a large periplasmic space (7–9) and an outer membrane (OM). However, this OM lacks lipopolysaccharide and common phospholipids typical of Gram-negative bacterial OMs, and instead has a lipid composition similar to the IM (10). The *D. radiodurans* OM is also covered by a regularly spaced, hexagonal surface layer or S-layer (11, 12). Previous studies have suggested that the S-layer is made of a protein called hexagonally packed intermediate-layer (HPI) surface protein (3, 8, 11, 13–17), while newer studies have suggested that a heterocomplex with gating properties, termed the S-layer deinoxanthin-binding complex (SDBC), forms a large part of the *D. radiodurans* cell envelope, including the S-layer (18, 19). A previously identified abundant protein called SlpA (UniProtKB Q9RRB6) is suggested to be the main component of this complex. Recently, an 11-Å resolution structure of this complex was reported using electron cryomicroscopy (cryo-EM), showing how it exhibits a triangular base partly embedded in the OM and a stalk departing orthogonally from the base, presumably away from the membrane (18). Deletion of *slpA* leads to substantial disruption of the *D. radiodurans* cell envelope, suggesting its important role in the maintenance of cell envelope integrity (20). Finally, it has been shown using biochemical experiments that the N-terminus of *D. radiodurans* SlpA binds to the PG-containing cell wall, demonstrating that at least the N-terminal segment of the molecule resides in the periplasmic space (21).

**Fig. 1.**
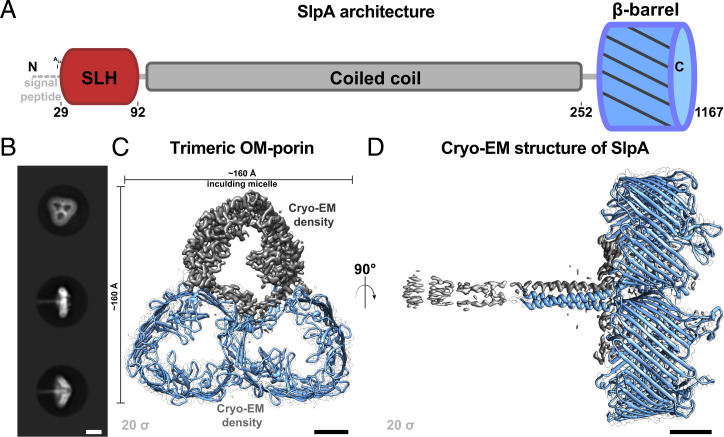
Cryo-EM reconstruction of *D. radiodurans* SlpA. (*A*) The SlpA protein contains a tripartite structure, including an N-terminal SLH domain, which is connected to a C-terminal β-barrel by a long coiled-coil segment. (*B*) Two-dimensional class averages of the trimeric SlpA specimen used for cryo-EM structure determination. Characteristic top and side views are shown. (*C*) Density map of the SlpA trimer (contour level on the *Lower Left*) shown from the *Top*. The resolution of the OMBB portion of the map is 2.9 Å, and resolution decreases toward the N-terminus, with a global resolution of 3.3 Å. Two subunits are shown as blue ribbons inside white envelope outlines and one as gray density (model hidden). Distance measurement includes the micelle density. (*D*) An orthogonal view of *C*, with the SlpA trimer shown from the side. The extended coiled coil degrades in resolution toward the N-terminus (see also *SI Appendix*, Fig. S1), presumably due to flexibility of the long stalk. (Scale bars in *B*, 100 Å; in *C* and *D*, 25 Å.)

In addition to the experimental observations introduced above, from an evolutionary perspective, an ortholog of *D. radiodurans* SlpA (UniProtKB Q5SH37) has also been characterized from the closely related thermophilic model bacterium *Thermus thermophilus* (22, 23). In line with data from *D. radiodurans*, deletion or truncation of *slpA* from *T. thermophilus* leads to remarkable disruption of the cell envelope (20, 24), underpinning its importance in cell surface organization. At the sequence level, SlpA contains a signal peptide, an SLH domain, a long, predicted α-helical region, and a C-terminal β-strand–rich domain, which is thought to fold into an OM β-barrel (18, 19) (Fig. 1*A*). Due to the presence of the N-terminal SLH domain, which commonly attaches S-layer proteins (SLPs) (23, 25–28) of Gram-positive bacteria to PG-linked pyruvylated secondary cell wall polymers (SCWPs), it has been suggested that SlpA constitutes the S-layer. Conversely, in *T. thermophilus*, SlpA has been shown to interact with PG through its SLH domain, suggesting a role for it as a periplasmic spacer (29). The role of SlpA in organizing the cell envelope of *D. radiodurans* and related deep-branching bacteria such as *T. thermophilus* is, therefore, still enigmatic.

In this study, we report the cryo-EM structure of the SlpA protein complex from *D. radiodurans.* Our structure shows that SlpA exhibits a tripartite organization, with its C-terminal part forming a homotrimeric 30-stranded OM β-barrel (OMBB), its central part forming a trimeric coiled coil that can traverse the large periplasmic space, and the extreme N-terminal part forming an SLH domain trimer that can interact with the PG layer. Our structure- and sequence-based bioinformatic analyses further show the presence of SlpA-like proteins in several phyla of phylogenetically deep-branching Gram-negative bacteria. Finally, combining our atomic structures and bioinformatic results with microscopy of wild-type and mutant cells, we report a model for the cell envelope of *D. radiodurans,* showing how this Gram-negative (diderm) bacterial SlpA protein shares several characteristics commonly found in Gram-positive (monoderm) SLPs, with connotations on prokaryotic evolution.

## Results

### Overall Structure of the *D. radiodurans* SlpA Complex.

To understand the molecular details of SlpA, we utilized previously described techniques (18, 19) to purify SlpA from *D. radiodurans* using detergent solubilization (*SI Appendix*, Fig. S1 and *Materials and Methods*). Cryo-EM images of the purified specimen showed single particles on the grid (*SI Appendix*, Fig. S1), which appeared to be made up of trimeric densities (Fig. 1*B*), as reported previously (18). We performed single-particle analysis on this cryo-EM data to solve a global 3.3-Å resolution structure of SlpA, with the best resolution in the β-barrel of 2.9 Å at the core (Fig. 1 *C* and *D* and *SI Appendix*, Fig. S1 and Table S1). The structure showed that SlpA forms a homotrimer of 30-antiparallel–stranded β-barrels (30 β-strands per SlpA monomer). The SlpA β-barrel is the first structurally characterized 30-stranded barrel and one of the largest single-chain β-barrels observed (30, 31). Since the SlpA complex was stabilized in detergent, and because the SlpA protein sequence possesses a β-signal motif (*SI Appendix*, Fig. S2), which is important for efficient targeting of OMBBs to the β-barrel assembly machine (BAM) complex (32), it is highly likely that the β-barrel is present in the OM of *D. radiodurans*, in line with previous results on *slpA* deletion mutants in *D. radiodurans* (20).

Bioinformatic analyses revealed that homologs of SlpA are widespread in the Deinococcus-Thermus phylum, with some species, such as *Deinococcus wulumuqiensis* and *T. thermophilus*, even possessing two copies of SlpA (*SI Appendix*, Table S2). The OMBB domain represents the most divergent part of SlpA proteins and contains either 28 or 30 β-strands depending on the species (*SI Appendix*, Fig. S3 and Table S2). For example, while the SlpA OMBB of *D. radiodurans, D. wulumuqiensis*, *Oceanithermus desulfurans*, and *Marinithermus hydrothermalis* possesses 30 strands, the SlpA OMBB of *T. thermophilus*, *Meiothermus ruber*, and *Deinococcus ficus* possesses 28 strands (*SI Appendix*, Table S2). The C-terminal OMBB of the *D. radiodurans* SlpA is preceded by a long, homotrimeric coiled-coil segment, which, in our cryo-EM map, is well resolved from residue 215 onwards. Together, there are extensive protein:protein homotrimeric interfaces both in the β-barrel and in the coiled-coil segment that appear to stabilize the trimeric SlpA complex (Fig. 1 *C* and *D*).

### SlpA Contains a β-Barrel with Several Insertions and a Coiled-Coil Stalk That Connects the OM to PG.

**Fig. 2.**
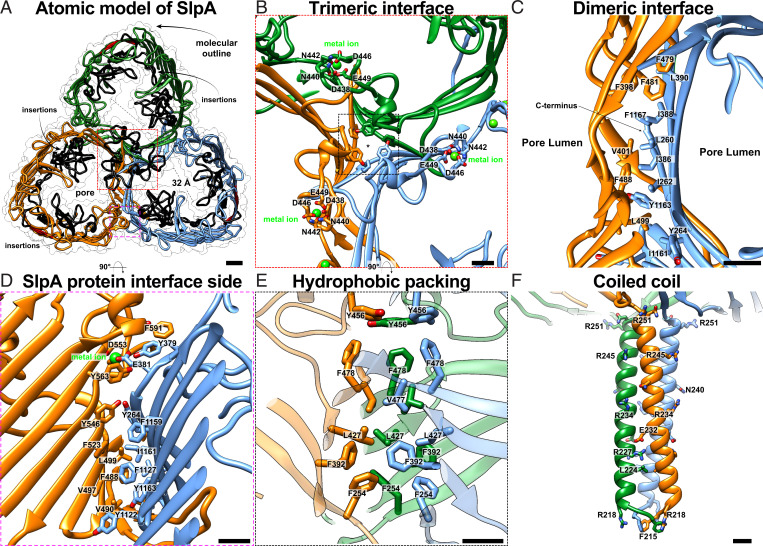
Atomic structure of the trimeric SlpA protein. (*A*) The refined atomic model of the trimeric SlpA protein shown as a ribbon diagram. The pore contains several insertions (black ribbons). (*B*) Closeup view of the central trimeric interface is shown where a typical insertion including metal-binding sites is found. (*C* and *D*) The dimeric SlpA:SlpA interface is lined by hydrophobic residues and stabilized by a metal-binding site (*SI Appendix*, Fig. S4). (*E*) The central trimeric interface is stabilized by hydrophobic packing of aromatic residues as shown in an orthogonal, magnified view of *B*. (*F*) Closeup view of the end of the coiled-coil segment. (Scale bars in *A*, 10 Å; in *B–F*, 5 Å.)

When compared to its homologs, the OMBB of *D. radiodurans* SlpA (residues 254 to 1167) contains several insertions positioned within the pore (Fig. 2*A* and *SI Appendix*, Fig. S3). Residues 272 to 377 form the most extensive, ordered insertion that lines the cavity of the pore. This insertion appears to be stabilized by a canonical bacterial SLP metal ion–binding site (33) coordinated by residues D274, D276, and D310 (*SI Appendix*, Fig. S4). Likewise, large insertions within the lumen of the pore, with putative metal ion–binding sites, are found between residues 429 to 471 and 686 to 753 (Fig. 2*B* and *SI Appendix*, Fig. S4). Although density for metal ions is resolved in our cryo-EM map (*SI Appendix*, Fig. S4), the identity of the metal is unknown. Sequence analysis reveals that these insertions are only present in closely related Deinococcales (e.g., *D. wulumuqiensis*) and are less extensive or absent in SlpA proteins of most other Deinococcales and Thermales (*SI Appendix*, Fig. S3), suggesting that SlpA of *D. radiodurans* may not fulfill a role as a pore, but rather functions as an abundant membrane scaffold organizing the cell envelope. Also, since many bacteria in the Deinococcus-Thermus phylum do not possess an S-layer built of the HPI protein, the extensive insertions in *D. radiodurans* may also be involved in anchoring the HPI-based S-layer in a substoichiometric manner, as previously suggested (18).

The protein:protein interface of the SlpA β-barrel consists mainly of stacked β-sheets from apposing barrels (Fig. 2 *C* and *D* and *SI Appendix*, Fig. S4). These sheets contain a hydrophobic patch made up of residues L260, I262, Y264, I386, I388, L390, and F392 stacking onto V401, F479, F481, F488, and L499 from the next subunit (Fig. 2 *C* and *D*). At the trimeric interface (C3 axis), another set of hydrophobic residues F254, F392, L427, V477, and F478 stabilize the complex (Fig. 2*E*).

**Fig. 3.**
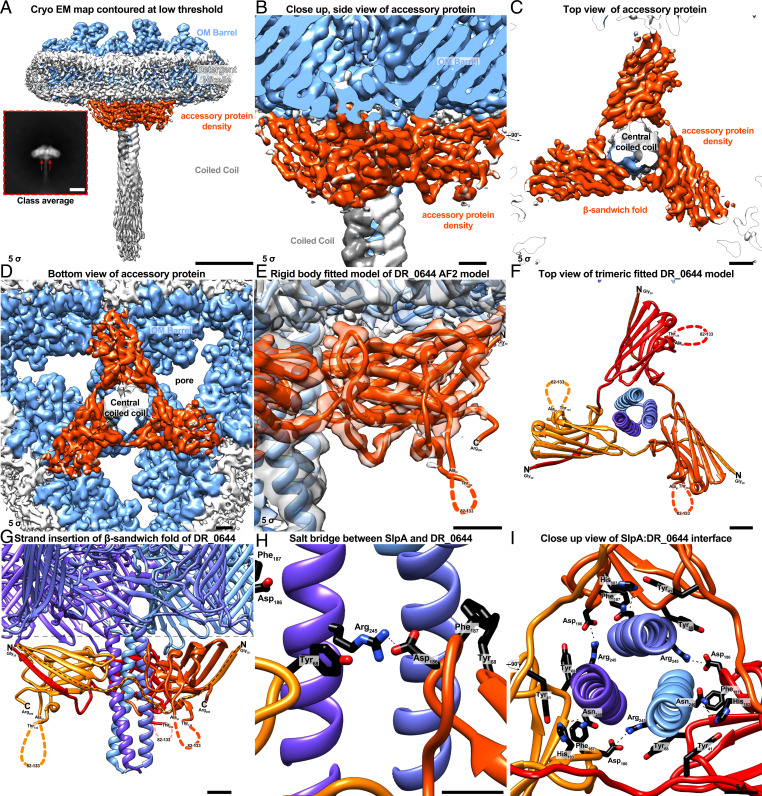
Additional protein density bound to SlpA. (*A*) Density map of SlpA OMBB trimer at a contour level of 5 σ shown in light blue embedded in a detergent micelle (white). An additional protein density (orange) is partially resolved, present in a subclass of particles seen in 2D class averages. (Scale bars: 50 Å; in *Inset*, 100 Å.) (*B–D*) Closeup view of the additional density seen from the *Side B*, *Top C*, and *Bottom D*. The accessory protein adopts a trimeric β-sandwich fold which is aligned with the OMBB without occluding the pore region. (*E*) Rigid body fit of the AlphaFold2 (37) (AF2) model of the uncharacterized protein DR_0644, which has been previously identified to be associated with SlpA (34), into the extra density not explained by SlpA. The model of the protein fits exceptionally well from the mature N-terminus (N, Gly_20_) to the C-terminus (C, Arg_206_), with the exception of an unstructured loop (82 to 133), which is in agreement with its low confidence of the pLDDT as measured by AF2. (*F* and *G*) The β-sandwich fold of the trimeric DR_0644 (red, orange-red, and orange ribbons) is completed by strand insertion of the first β-strand of the next clockwise oriented subunit, as seen from the *Top F* and *Side G*. (*H* and *I*) The interface of the SlpA:DR_0644 is stabilized by a prominent salt bridge between SlpA R245 and DR_0644 D186 and further protein:protein interactions such as hydrogen bonding between SlpA N240 and DR_0644 H183. (Scale bars in *B–G*, 10 Å; in *H* and *I*, 5 Å.)

There is clear density in the map for residues 215 to 253 that make up the coiled-coil segment connected with the OMBB (Fig. 2*F* and *SI Appendix*, Figs. S4*F* and S1 *E* and *F*). The coiled coil consists of a highly conserved, prominent salt bridge between E232 and R227 (Fig. 2*F* and *SI Appendix*, Fig. S4). Residue R245 points away from the axis of the coiled coil and is bound to a poorly resolved density for a protein rich in β-strands (Fig. 3). We were able to ascertain the identity of this protein using the map density combined with structural modeling (DR_0644; UniProt Q9RWM2); however, due to the weak density, atomic model refinement of this newly identified protein was not performed. This protein has been previously detected in a mass spectrometric study of the *D. radiodurans* cell envelope (34), supporting our structural identification. This accessory protein is predicted to be a lipoprotein by SignalP 6.0 (35), as it contains a type II signal peptide followed by a conserved cysteine, which is presumably posttranslationally lipid modified. Homologs of DR_0644 are found in many other Deinococcales, but are absent in Thermales. The residues of the SlpA coiled coil prior to residue 215 are less well resolved in our cryo-EM map (*SI Appendix*, Fig. S1 *E* and *F*), but diffuse density for the N-terminal part of the protein extends well beyond the well-resolved part of the coiled coil (Figs. 1*D* and 3*A*). This extended arrangement of the coiled coil supports SlpA’s role in bridging the OM, where the β-barrel (residues 254 to 1167) is situated, and the PG, which has been shown to bind to the N-terminal (predicted residues 29 to 92, *SI Appendix*, Fig. S2) SLH domain (21).

### Effect of *slpA* Deletion on the *D. radiodurans* Cell Envelope.

**Fig. 4.**
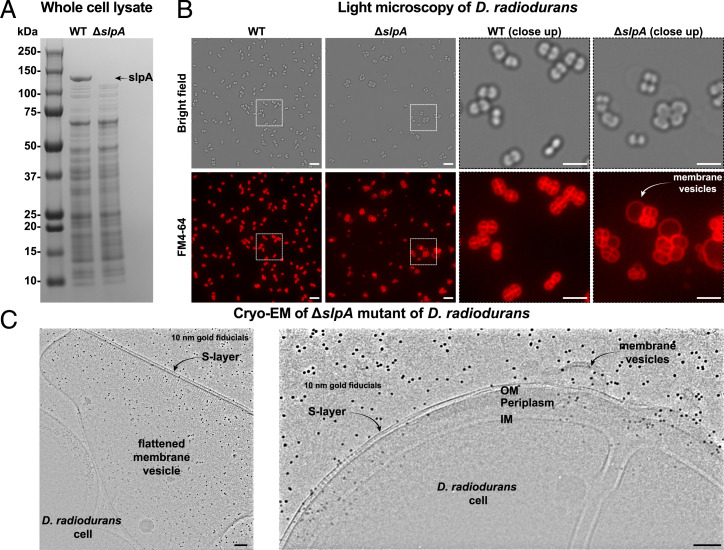
Effect of *slpA* deletion on *D. radiodurans* cells. (*A*) SDS-PAGE analysis of whole-cell lysates of wild-type (WT) and the Δ*slpA* mutant (20) show that SlpA is one of the most abundant proteins in the cell. We note that no Ethylene-diamine tetraacetic acid (EDTA) was added to the sample, which has been shown to be critical for visualizing HPI (16). (*B*) Optical microscopy of the two strains (membranes fluorescently stained with fn4-64) show large, membranous vesicular shedding from the Δ*slpA* mutant. (Scale bars: 10 µm full field of view; 5 µm closeup.) (*C*) Cryo-EM analysis of the Δ*slpA* mutant further shows disruption of the cell envelope, with cells and cell membranes coated with a substantial S-layer. (Scale bars: 100 nm.)

To verify our cryo-EM and bioinformatic data above, which strongly suggested that SlpA connects the OM to the PG layer, important for organizing the cell envelope of *D. radiodurans*, we studied the effect of deletion of *slpA* on the cell envelope. The whole-cell lysate of a Δ*slpA* mutant from a previous study (20) was compared with the whole-cell lysate of wild-type *D. radiodurans* cells (Fig. 4*A*). One of the strongest bands in the wild-type lysate, corresponding to SlpA (see also *SI Appendix*, Fig. S1*B* for the purified protein), was missing in the Δ*slpA* mutant, illustrating that SlpA is one of the most abundant proteins in the cell. To understand the effect of *slpA* deletion on the cell envelope of *D. radiodurans*, we subsequently performed optical microscopy of the two strains, with the cell membranes stained with a fluorescent dye (FM4-64, *Materials and Methods*). Compared to the wild-type strain, the Δ*slpA* cells showed large, membranous vesicular secretions in the culture that were associated with *D. radiodurans* cells (Fig. 4*B*). These membrane secretions strongly indicate that the cell envelope of *D. radiodurans* has been disrupted due to *slpA* deletion. To verify this further, we performed cryo-EM imaging of the Δ*slpA* cells and observed large disruptions in the OM, with several secreted OM vesicles coated with an S-layer, which was indistinguishable from a wild-type *D. radiodurans* S-layer (Fig. 4 *C*, *Left*). Interestingly, we also detected large patches of the S-layer on the surface of the Δ*slpA* cells with the same appearance as the wild-type S-layer (Fig. 4 *C*, *Right*), in agreement with previous work (20). Taken together, analysis of the Δ*slpA* cells shows that SlpA is essential for maintaining the integrity of the OM of *D. radiodurans*; however, it is not essential for the assembly of the S-layer on cells and cell membranes.

### Structural Modeling of the Periplasmic Part of the *D. radiodurans* SlpA.

Next, we used the power of the recently developed, state-of-the-art structure prediction method AlphaFold-Multimer (36), which has been shown to yield fairly accurate atomic models of homo- and heteromeric complexes (37), to model the periplasmic part of *D. radiodurans* SlpA. This part of the sequence (residues 20 to 252), comprising the SLH domain and a section of the coiled coil, was poorly resolved in our map, presumably due to its dynamic nature. The model yielded by AlphaFold-Multimer had high per-residue confidence (predicted local-distance difference test [pLDDT]) and low predicted aligned error (PAE) values, both of which are indicators for high accuracy of the model (*SI Appendix*, Fig. S5). In fact, the part of *D. radiodurans* homotrimeric coiled-coil segment resolved in our cryo-EM map (residues 215 to 254) and the corresponding part in the AlphaFold-Multimer model showed remarkable similarity, superimposing with a rmsd of ∼0.43 Å over all Cα atoms. The complete model of the N-terminal part of *D. radiodurans* SlpA shows that the length of the homotrimeric coiled-coil segment is ∼28 nm, which is in good agreement with our measurements from cryo-EM (*SI Appendix*, Fig. S8, ∼29 nm). The coiled-coil segment exhibits two β-layers (*SI Appendix*, Fig. S2), which are triangular supersecondary structural elements formed in trimeric coiled coils to compensate for local strains resulting from the insertion of two or six amino acids into the canonical heptad repeats (38).

**Fig. 5.**
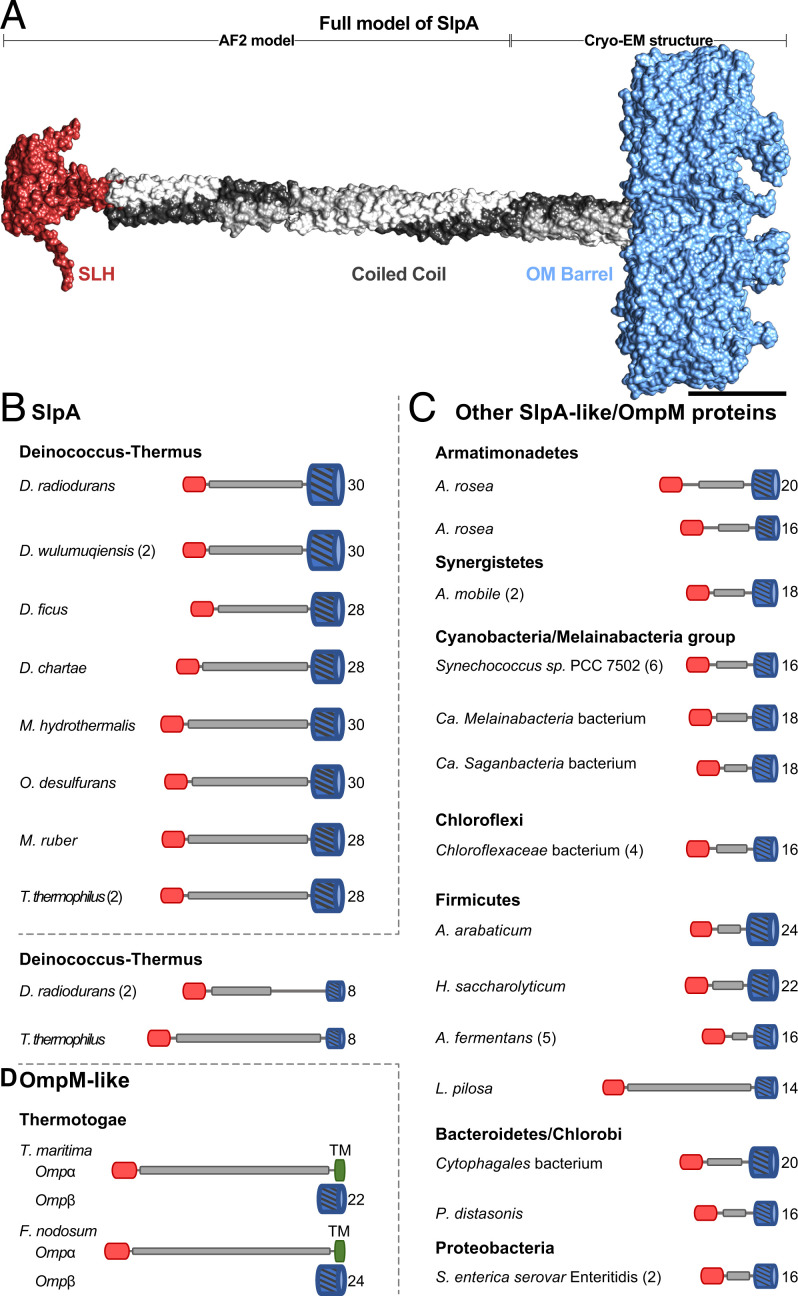
Structural modeling of SlpA-like proteins reveals common organizational principles of OM–PG connectors. (*A*) Combined cryo-EM and AlphaFold model of SlpA from *D. radiodurans.* (Scale bar: 50 Å.) (*B–D*) The domain organization of representative SlpA and SlpA-like/OmpM proteins from bacteria of several Gram-negative phyla are shown. While they exhibit a shared tripartite organization, comprising an N-terminal SLH domain, a central coiled-coil segment, and a C-terminal OMBB, the length of the coiled-coil segment and the number of β-strands (indicated on the *Right* end of the cartoons) in the OMBB is quite varied. Some organisms, such as *D. wulumuqiensis* and *T. thermophilus*, contain two or more paralogs (indicated within parentheses). Species of the phylum Thermotogae, e.g., *T. maritima* and *Fervidobacterium nodosum*, contain two highly abundant SlpA-like OM proteins Ompα and Ompβ. While Ompβ is an OMBB, Ompα contains an N-terminal SLH domain followed by a long coiled-coil stalk and a C-terminal transmembrane helix (TM, dark green). Accession details for the shown proteins are provided in *SI Appendix*, Table S2.

Next, we analyzed the periplasmic segments of several other SlpA proteins at an organizational level (Fig. 5). The length of the coiled-coil segment of SlpA is comparably long in other Deinococcales and is even longer in Thermales (Fig. 5 and *SI Appendix*, Fig. S6), supporting a role for SlpA as an OM–PG connector or spacer. Moving toward the IM, the coiled-coil segment is connected to the SLH domain via a short, disordered linker in *D. radiodurans* (*SI Appendix*, Fig. S2). The SLH domain of SlpA, like other previously characterized SLH domains (39), is also predicted to form a trimer. The SLH domain is highly conserved among Deinococcus-Thermus SlpA proteins (*SI Appendix*, Fig. S7), with an average pairwise sequence identity of greater than 60% and conserved sequence motifs (W, residue 41; GVILG, residues 53 to 57; and TRYE, residues 70 to 73 in *D. radiodurans* SlpA) characterized to be important for interactions with PG-linked SCWPs in other SLH domains, such as the SLH domains of *Paenibacillus alvei* S-layer protein SpaA (26, 39–41). This agrees with our cryo-EM data and previous biochemical experiments (21), demonstrating that the *D. radiodurans* SLH domain connects the OMBB to the PG at the N-terminal end of the coiled coil.

### Several OMBB Proteins in Deep-Branching Gram-Negative Bacteria Contain Coiled Coils Connected to SLH Domains.

To investigate the presence of other SlpA-like proteins in *D. radiodurans,* we searched for all OMBB-containing proteins encoded in its genome using the predictive power of HHpred (42) and AlphaFold (37). In addition to SlpA, we found 19 additional OMBB-containing proteins, with three predicted to form large, 38-stranded β-barrels (*SI Appendix*, Table S3). Curiously, similarly to SlpA, two of these 19 OMBB proteins also contain a central coiled-coil segment and an N-terminal SLH domain possessing residues important for binding PG-linked SCWPs (Fig. 5 and *SI Appendix*, Fig. S7). However, unlike SlpA, they comprise an 8-stranded β-barrel that is reminiscent of the β-barrel of the outer membrane protein A (OmpA) (43), which is an OM–PG tether found in some Gram-negative bacteria such as *Escherichia coli*. Homologs of these two SlpA-like *D. radiodurans* proteins are widespread in the Deinoccocus–Thermus phylum, suggesting that they, like SlpA, might also be involved in connecting the OM to the inner cell envelope. The presence of several OMBBs as well as the recently described PilQ secretin complex that traverses both membranes (34), suggests that even though SlpA is a highly abundant molecule in the *D. radiodurans* cell envelope, it cannot fully tile the OM and is probably not an integral part of the S-layer. We, however, cannot rule out that it may play a substoichiometric, minor role in anchoring the HPI protein.

We next investigated whether SlpA-like proteins are also present in other phylogenetically deep-branching Gram-negative bacterial lineages because their presence could represent an ancestral mechanism for tethering the OM to the inner cell envelope. In fact, a recent study carried out a systematic search for OM–PG connectors across many bacterial genomes and found that SlpA-like connectors are widespread in diderm Terrabacteria, with the proposal that they represent the main OM–PG tethering system in this clade (44). Terrabacteria is a large clade that includes both monoderm and diderm phyla, such as Deinococcus-Thermus, Synergistetes, Cyanobacteria, and Firmicutes. Furthermore, this study classified SlpA-like proteins as OmpM proteins based on the name given to the SlpA-like protein in *Selenomonas ruminantium* (45, 46). Concomitantly, we also detected a widespread occurrence of SlpA-like/OmpM proteins in several deep-branching phyla of Terrabacteria, including Synergistetes, Cyanobacteria, Chloroflexi, Chlorobi, Candidatus Melainabacteria, Armatimonadetes, and Bacteroidetes (Fig. 5) and in the Gram-negative lineages Halanaerobiales, Negativicutes, and Limnochordia of the largely Gram-positive phylum Firmicutes. SlpA-like/OmpM proteins are frequently annotated as iron uptake porin, carbohydrate porin, S-layer protein, S-layer homology domain-containing protein, or hypothetical protein in protein sequence databases. However, in the cyanobacterium *Synechococcus* PCC 6301 (47) and the Negativicutes *S. ruminantium* (45, 46, 48, 49) and *Veillonella parvula* DSM2008 (44, 50), SlpA-like proteins have been shown to be highly abundant in the OM and important for maintaining the integrity of the OM. Although SlpA-like/OmpM proteins exhibit a tripartite domain organization as SlpA of *D. radiodurans* and possess sequence motifs important for interactions with PG-linked SCWPs in their SLH domain, they contain OMBBs of varying sizes and much shorter coiled-coil segments (Fig. 5), which is expected, given that the periplasmic space in *D. radiodurans* is substantially thicker compared to most Gram-negative bacteria. We predict that like SlpA, other OmpM proteins probably also form homotrimeric complexes that link the OM to the inner envelope.

### Model of the *D. radiodurans* Cell Envelope.

To relate our atomic structural and bioinformatic data with the native cell envelope, we next collected electron cryotomograms of whole *D. radiodurans* cells and envelopes of partly lysed cells (*SI Appendix*, Fig. S8). In line with previous reports (9, 11), we observed a cell envelope with two membranes, a large periplasmic space of 121 ± 4 nm, and a thick PG layer. As expected from our structural results, we observed a fuzzy density corresponding to the start of the wide PG layer at a distance of 30 ± 3 nm from the OM, in agreement with the length of the periplasmic coiled-coil segment of SlpA observed in our atomic model (28 nm) and class averages (∼29 nm, *SI Appendix*, Fig. S8). Outside the OM, we observed that the S-layer was positioned 18 ± 1 nm away from the OM, with clear repeating subunits observed within this density (*SI Appendix*, Fig. S8).

**Fig. 6.**
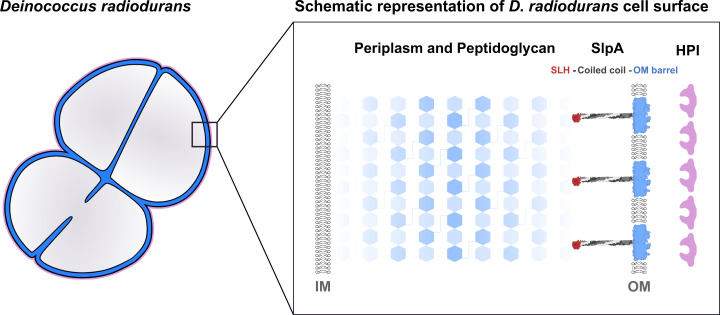
Model of the *D. radiodurans* cell envelope. Schematic model of the *D. radiodurans* cell envelope shows how SlpA connects the OM to the PG layer via long coiled coils and an N-terminal SLH domain, placing data from previous studies into context, and providing a structural framework for understanding the cell envelope of phylogenetically deep-branching Gram-negative bacteria.

Taken together, we report an updated model of the *D. radiodurans* cell surface (Fig. 6). We suggest that SlpA does not tile the OM, supported by the presence of several other OMBBs in the *D. radiodurans* genome, and neither is it an integral part of the S-layer, because deletion of *slpA* left the *D. radiodurans* S- layer unchanged compared to wild-type cells (Fig. 4). The HPI S-layer could be held in a substoichiometric manner by OMBBs of SlpA, which are present in abundance in the OM. Finally, SlpA OMBBs are additionally connected through coiled-coil segments to the PG layers via SLH domains, and biochemical binding of the SlpA SLH domain to PG has been shown previously (21). These multiple interactions within the cell surface show an important role of SlpA in organizing the *D. radiodurans* cell envelope.

## Discussion

In this study, we present structural data to resolve a long-standing conundrum about the role of the OM protein SlpA in organizing the cell surface of *D. radiodurans*. While initial studies suggested that the S-layer of *D. radiodurans* is built by the HPI protein, more recent studies have proposed that it is formed of multiple proteins, including HPI and SlpA. Our results indicate that SlpA cannot fully tile the OM and that it is not a fundamental component of the S-layer, but that it connects the OM to the PG layer by forming extended homotrimers. SlpA might play a minor, substoichiometric role in anchoring the HPI protein, although this role of SlpA has not been demonstrated. SlpA exhibits a tripartite organization, comprising an OMBB trimer embedded in the OM, a long coiled-coil stalk, and an SLH domain trimer, typically found in Gram-positive SLPs (25). Combining our atomic structures and bioinformatic results with tomography of native cell envelopes, we report an updated model for the complex, multilayered cell envelope of *D. radiodurans* (Fig. 6), which will serve as a structural framework for understanding the cell surface of similar deep-branching bacteria with atypical envelopes.

Furthermore, we show that SlpA-like/OmpM proteins, frequently containing OMBBs of varying sizes and coil-coiled segments of varying lengths, are widespread in the Deinococcus-Thermus phylum as well as in several phyla of deep-branching Gram-negative bacteria, suggesting that they represent an ancestral mechanism for stabilizing the cell envelope by connecting the OM to the PG layer. In some Proteobacteria, such as *E. coli*, *Coxiella burnetii*, *Pseudomonas aeruginosa*, and *Legionella pneumophila*, highly abundant OM proteins that form covalent or noncovalent connections between the OM and the PG layer have been shown to be important for the stabilization and the spacing of the OM with respect to the IM. Such proteins include Braun’s lipoprotein (Lpp) (51, 52); PG-associated lipoprotein (Pal) (53); and the OMBB proteins OmpA (43, 53), OprF (54, 55), and BbpA (56). However, OM–PG connectors remain poorly characterized in most other phyla of Gram-negative bacteria (44). The physiological role of SlpA-like proteins has been studied only in a handful of species thus far (24, 44, 57), but we speculate that they may be involved in maintaining the integrity of the OM in several phyla of Gram-negative bacteria, as triple Δ*ompM1-3* mutants of *V. parvula* share a similar phenotype of disrupted cell envelopes and severe growth defects compared to Δ*slpA D. radiodurans* mutants (20, 44).

We also expect that the multidomain architecture of SlpA is crucial for its role as an organizational spacer in the cell envelope. To illustrate this, in the same manner as *D. radiodurans*, the deep-branching hyperthermophilic bacterium *Thermotoga maritima* also exhibits an unusual cell envelope that is thought to be stabilized by two equally abundant OM proteins, Ompα and Ompβ, which resemble SlpA (Fig. 5) (58, 59). While Ompα has been characterized to be a rod-shaped spacer in electron micrographs, Ompβ forms triangular, porin-like assemblies in the OM. Like SlpA, Ompα contains an N-terminal SLH domain and a long coiled-coil segment, which has been predicted to be 45 nm in length; however, instead of an OMBB, the C-terminal end of Ompα contains a transmembrane helix that anchors it to the OM. The identity of Ompβ has not been established experimentally yet, but an OMBB, encoded by a gene that occurs adjacent to the gene encoding Ompα in an operon in *T. maritima* and some closely related organisms, is likely to be Ompβ (60), and this OMBB is predicted by AlphaFold to contain 22 β-strands (Fig. 5*D*). We speculate that, like SlpA, Ompα and Ompβ associate to form a homotrimeric complex, a scenario that would be consistent with both the OMBB and the coiled-coil part of SlpA-like proteins being important for acting as a spacer, critical for organizing the cell envelope. Moreover, while *T. maritima* contains two further paralogs of Ompα, which have also been implicated to play a role in the organization of its cell envelope (61), orthologs of Ompα and Ompβ are widespread in the phylum Thermotogae (44, 60).

Questions of whether monoderm or diderm bacteria came first, and how and when the transition between them occurred, are major open questions in evolutionary biology (62–64). The widespread occurrence of the SLH domain in monoderm and diderm bacterial proteins, including SLPs and SlpA-like proteins, suggests that the SLH domain was established as a PG-binding domain very early in the evolution of bacteria (65). Furthermore, given the widespread occurrence of SlpA-like/OmpM proteins in diderm bacteria and the role of SlpA in organizing the OM of *D. radiodurans*, it appears plausible that an ancestral SlpA-like protein was already present and functioned as an OM–PG connector in the common ancestor of diderm bacteria (44). In recent years, an ever-increasing body of evidence has supported the hypothesis that the last common ancestor of all bacteria already possessed a diderm envelope and that monoderms arose from diderms by the loss of the OM (44, 63, 64). Therefore, it is tempting to speculate that SLH domain–containing SlpA-like proteins may have facilitated the loss of the OM during the transition between monoderm and diderm bacteria (44), and it would be fascinating to explore this possibility moving forward.

## Materials and Methods

### SlpA Protein Purification.

*D. radiodurans* cells from American Type Culture Collection (ATCC BAA816) were grown in modified tryptone-glucose-yeast extract (TGY) medium supplemented with 5 µM MnCl_2_ (66). For protein purification of wild-type SlpA, 4 L of modified TGY medium were inoculated 1:25 with a late-log phase preculture, and cells were grown overnight with shaking at 30 °C. Cells were harvested by centrifugation (5,000 relative centrifugal force [rcf], 4 °C, 30 min), and the cell pellet was resuspended in 50 mL lysis buffer (100 mM Tris/HCl pH 8.0, 150 mM NaCl, 5 mM MgCl_2_, 50 µg/mL DNaseI, 1 U/mL benzonase [Sigma-Aldrich], 1× cOmplete protease inhibitor [Roche]) per 1 L cell pellet. Cells were lysed by passing the suspension five times through a homogenizer at 22,500 pounds per square inch (psi), and unlysed cells were removed by centrifugation (2,000 rcf, 4 °C, 15 min). Remaining cell debris was isolated by centrifugation (48,000 rcf, 4 °C, 30 min). To degrade PG, the pellet was resuspended in 40 mL lysozyme buffer (100 mM Tris/HCl pH 8.0, 500 µg/mL lysozyme, 1× cOmplete inhibitor) and incubated on a rotary wheel for 16 h at 4 °C. The remaining insoluble fraction was pelleted by centrifugation (48,000 rcf, 4 °C, 30 min), washed three times with 37.5 mL wash buffer (100 mM Tris/HCl pH 8.0, 150 mM NaCl), and separated by centrifugation after each step (48,000 rcf, 4 °C, 30 min). Membrane proteins in the final washed pellet were resuspended in 40 mL buffer (20 mM Tris/HCl pH 8.0) and extracted with detergent by adding dropwise a 10% (wt/vol) stock solution of n-dodecyl β-D-maltoside (DDM, Anatrace) to a final concentration of 1.3% (wt/vol). The protein suspension was next incubated on a rotary wheel for 3 h at 4 °C and nonsolubilized material was removed by centrifugation (30,000 rcf, 4 °C, 30 min). The protein solution was then loaded onto equilibrated 5-mL HiTrap-Q columns (GE Healthcare) using an ÄKTA pure 25 system (GE Healthcare), and unbound protein was washed away with 50 mL binding buffer (20 mM Tris/HCl pH 8.0, 0.05% [wt/vol] DDM). Bound protein was eluted with an increasing gradient of 75 mL elution buffer (20 mM Tris/HCl pH 8.0, 0.05% [wt/vol] DDM, 1 M NaCl). Fractions containing SlpA were pooled, concentrated using a 30-kDa molecular weight cutoff (MWCO) Ultra Centrifugal tube (Amicon), and loaded to a Superose 6-Increase 10/300 GL column (GE Healthcare) equilibrated with 20 mM Hepes/NaOH pH 7.5, 150 mM NaCl, 0.02% (wt/vol) DDM. Protein was eluted in the same buffer, and fractions containing SlpA were collected, concentrated (Amicon 30-kDa MWCO) to 200 µL, and then dialyzed against 100 mL Size Exclusion Chromatography (SEC) buffer (20 mM Hepes/NaOH pH 7.5, 150 mM NaCl, 0.02% [wt/vol] DDM) for 2 h with a 10-kDa MWCO cutoff. For cryo-EM grid preparation, the final protein solution was then concentrated to 4.45 mg/mL and immediately used. Purified SlpA was kept at 4 °C, reloaded onto a Superose 6 Increase 10/300 GL column (GE Healthcare), and analyzed by sodium dodecyl sulfate-polyacrylamide gel electrophoresis (SDS-PAGE), which showed minimal degradation upon prolonged storage. Whole-cell lysate of *D. radiodurans* ATCC 13939 wild-type and *D. radiodurans* Δ*slpA* mutant cells (20) were prepared by lysing an equivalent amount of cells in reducing SDS-PAGE loading buffer at 95 °C for 10 min before SDS-PAGE analysis. Chromatograms and SDS-PAGE gel images were visualized with MATLAB (MathWorks) and Fiji (67), respectively.

### Cryo-EM Sample Preparation.

For cryo-EM grid preparation procedures described previously were followed (68). Briefly, 2.5 µL of 4.45 mg/mL SlpA sample or sonicated (5 s, 15 mA amplitude) late-log cultures *D. radiodurans* culture were applied to a freshly glow discharged Quantifoil R2/2 Cu/Rh 200 mesh grid, adsorbed for 10 s, blotted for 5 s, and plunge frozen into liquid ethane in a Vitrobot Mark IV (Thermo Fisher), while the blotting chamber was maintained at 100% humidity at 10 °C. For electron cryotomography (cryo-ET), 10 nm protein-A gold (Cell Microscopy Core [CMC] Utrecht) was additionally added to the samples immediately prior to grid preparation.

### Cryo-EM Data Collection and Single-Particle Analysis.

For data collection, single-particle cryo-EM data on purified SlpA protein were collected as described previously (68, 69). In short, a Titan Krios G3 microscope (Thermo Fisher) was used, operating at 300 kV fitted with a Quantum energy filter (slit width 20 eV) and a K3 direct electron detector (Gatan) with a sampling pixel size of 0.546 Å running in counting superresolution mode. For the SlpA specimen, a total of 2,294 movies were collected in two sessions with a dose rate of 2.98 e^−^/pixel/s on the camera level. The sample was subjected to 4.8 s of exposure, during which a total dose of 47.909 e^−^/Å^2^, respectively, was applied, and 40 frames were recorded per movie (*SI Appendix*, Table S1).

Image processing, as detailed in previous single-particle cryo-EM studies from our laboratory (68, 69), was carried out by clustering the raw movies into optics groups based on the XML metadata of the data-collection software EPU (Thermo Fisher) using a *k*-means algorithm implemented in EPU_group_AFIS (https://github.com/DustinMorado/EPU_group_AFIS). The clustered movies were motion corrected, dose weighted, and Fourier cropped (2×) with MotionCor2 (70) implemented in RELION3.1 (71). Contrast transfer functions (CTFs) of the radiation-induced motion-compensated micrographs were estimated using CTFFIND4 (72). Initially, micrographs were denoised using TOPAZ (73) using the UNET neural network and 2,893 particles were manually picked. Particle coordinates were used to train TOPAZ picker (74) in 5× downsampled micrographs with the neural network architecture ResNet8 and picked particles were extracted in 4× downsampled 128 × 128 boxes and classified using reference-free two-dimensional (2D) classification inside RELION3.1, a strategy also described in detail in our previous studies (68, 69). An initial subset of 76,119 particles was then used to retrain TOPAZ, followed by another round of particle extraction and reference-free 2D classification. Particles belonging to class averages with high-resolution features were combined, and duplicate particles within 100 Å where removed, and an initial model was generated with 4× downsampled particles in 128 × 128 boxes using the stochastic gradient descent (SGD) algorithm within RELION3.1. The initial reference was aligned to the C3 symmetry axis and the merged particle subset was reextracted in 512 × 512 boxes and subjected to a focused three-dimensional (3D) autorefinement on the central porin and the first heptad of the coiled coil using the rescaled and blurred (30-Å lowpass filtered) output from the symmetry aligned initial model. Per-particle defocus, anisotropy magnification, and higher-order aberrations (71) were refined inside RELION-3.1, followed by signal subtraction of the detergent micelle and another round of focused 3D autorefinement, as described in our previous studies using single-particle cryo-EM (68). The reconstruction was further improved by Bayesian particle polishing (75), and a focused 3D classification without refinement of the poses. The final output map was obtained from 122,412 particles, which was postprocessed using a smooth mask focused on the trimeric OMBB, including the first heptad of the coiled coil with a global resolution of 3.25 Å, according to the gold standard Fourier shell correlation criterion of 0.143 (76). The cryo-EM map has been deposited at the Electron Microscopy Data Bank (EMD-15378), and single-particle data analysis statistics are summarized in *SI Appendix*, Table S1.

### Cryo-ET Data Collection, Tomogram Segmentation, and Subtomogram Averaging.

For tomographic data collection, the SerialEM software (77) was used as described previously (68, 69, 78). Tilt series data collection of cellular specimens was performed on the same Titan Krios microscope as above using the Quantum energy filter (slit width 20 eV) and the K3 direct electron detector running in counting mode. Tilt series (defoci ranging from −8 to −11 µm) were collected between ±60° in a dose symmetric scheme (79) with a 2° tilt increment. A cumulative dose of 121 e^−^/Å^2^ with a dose rate of 10.523 e^−^/px/s was applied during data collection, at a pixel size of 3.468 Å.

Tilt series alignment using gold fiducials and tomogram generation was performed in IMOD (80). Tensor voting–based membrane detection was performed with TomosegmemTV (81) and refined and visualized in University of California San Francisco (UCSF) Chimera (82) and UCSF ChimeraX (83). Distances between inner membrane, peptidoglycan layer, outer membrane, and S-layer were determined at multiple positions along the cell surface throughout the tomogram. Subtomogram averaging analysis of the *D. radiodurans* cell surface was performed using previously described methods (84, 85), also previously applied to Gram-negative bacterial cell surfaces (69).

### Model Building and Refinement.

The carbon backbone of the SlpA protein was manually traced through a single subunit of cryo-EM density using Coot (86). The atomic model was subjected to several rounds of refinement using REFMAC5 (87) inside the CCP-EM software suite (88) and PHENIX (89), followed by manually rebuilding in Coot and interactive refinement using ISOLDE (90) inside UCSF ChimeraX. Model validation was performed in PHENIX and CCP-EM, and data visualization was performed in UCSF Chimera and UCSF ChimeraX and PyMOL (Schroedinger LLC). The refined atomic model has been deposited in the Protein Data Bank (PDB ID 8AE1).

### Light Microscopy.

*D. radiodurans* Δ*slpA* mutant cells and the corresponding parental wild-type strain (ATCC 13939) were obtained from ref. 20 and grown in modified TGY medium overnight with shaking at 30 °C. For light microscopy 1 mL of cell cultures was harvested at 6,000× rcf for 5 min and cell pellets were resuspended in 1 mL 1× phosphate buffered saline (PBS). Cells were stained for 2 min on ice with fn4-64 (Invitrogen) by adding a freshly prepared stock solution (500 µg/mL) to a final working concentration of 10 µg/mL. Stained cells were immediately imaged with a 100× objective (0.75 numerical aperture) on an Axio Imager M2 (Zeiss) by adding 10 µL of cell suspension onto a glass slide and covering it with coverslip. Light microscopy images were analyzed with Fiji (67), by autocontrasting the entire field of view.

### Bioinformatic Analysis.

A structural model of the periplasmic, homotrimeric segment (residues 20 to 252) of *D. radiodurans* SlpA was built using an installation of AlphaFold-Multimer v2.2.0 (36) at the Max Planck Computing and Data Facility in Garching. The prediction was carried out in default settings, and the model (ranked_0.pdb) with the highest confidence was picked for further use (*SI Appendix*, Fig. S5). Homologs of *D. radiodurans* SlpA in the Deinococcus-Thermus phylum were detected using the National Center for Biotechnology Information (NCBI) BLAST Web server in default settings (91). To detect SlpA-like proteins in Gram-negative bacteria, we used a three-step approach. First, we searched the nonredundant protein sequence database at NCBI for homologs of the SLH domain of *D. radiodurans* SlpA; the search was restricted to Gram-negative phyla of bacteria. Next, we inspected the obtained sequences for the presence of a central coiled-coil segment using PCOILS (92) and a C-terminal OMMB using HHpred (93) searches against the ECOD (94) profile hidden Markov model (HMM) database. Finally, the three-dimensional structures of some representative SlpA-like proteins (*SI Appendix*, Table S2) were predicted using AlphaFold (37). To detect OMBB proteins in the proteome of *D. radiodurans*, we searched its profile HMM database with HHpred in the MPI Bioinformatics Toolkit (42). The searches were seeded with sequences of OMBBs from the ECOD X group “outer membrane meander beta-barrels.” Next, to analyze the domain composition and the number of β-strands in the barrel, we built structural models of the obtained matches using AlphaFold (*SI Appendix*, Table S2). Multiple sequence alignments of the SLH (*SI Appendix*, Fig. S7), coiled-coil (*SI Appendix*, Fig. S6), and OMMB (*SI Appendix*, Fig. S3) domains were calculated using PROMALS3D (95) and were subsequently curated manually based on AlphaFold models.

## Supplementary Material

Supplementary File

## Data Availability

All study data are included in the article and/or supporting information, reasonable requests for data will be fulfilled by Tanmay A.M. Bharat. The SlpA cryo-EM map has been deposited in the Electron Microscopy Data Bank (EMDB) with the accession code EMD-15378 (96) and the corresponding refined atomic model has been deposited in the Protein Data Bank (PDB) with the accession number 8AE1 (97). For further details see *SI Appendix*, Table S1.
