# Supplementary material for: A multidomain connector links the outer membrane and cell wall in phylogenetically deep-branching bacteria
Source: Proc Natl Acad Sci U S A. 2022 Aug 9;119(33):e2203156119. doi: 10.1073/pnas.2203156119 (PMC9388160; doi:10.1073/pnas.2203156119)
Supplement: Supplementary File [file pnas.2203156119.sapp.pdf]

PNAS
www.pnas.org

Supplementary Information for

A multi-domain connector links the outer membrane and cell wall in phylogenetically deep-branching bacteria

Authors

Andriko von Kügelgen[1,2], Sofie van Dorst[2], Vikram Alva[3,*] and Tanmay A. M. Bharat[1,2,*]

Affiliations

1 – Structural Studies Division, MRC Laboratory of Molecular Biology, Francis Crick Avenue, Cambridge CB2 0QH, United Kingdom

2 – Sir William Dunn School of Pathology, University of Oxford, Oxford OX1 3RE, United Kingdom

3 – Department of Protein Evolution, Max Planck Institute for Biology Tübingen, Max-Planck-Ring 5, Tübingen 72076, Germany

* **Correspondence to** Vikram Alva and Tanmay A.M. Bharat

Email: vikram.alva@tuebingen.mpg.de and tbharat@mrc-lmb.cam.ac.uk

This PDF file includes:

Figures S1 to S8

Tables S1 to S3

Legend for Movie S1

SI References

Other supplementary materials for this manuscript include the following:

Movie S1

Supplementary Figures

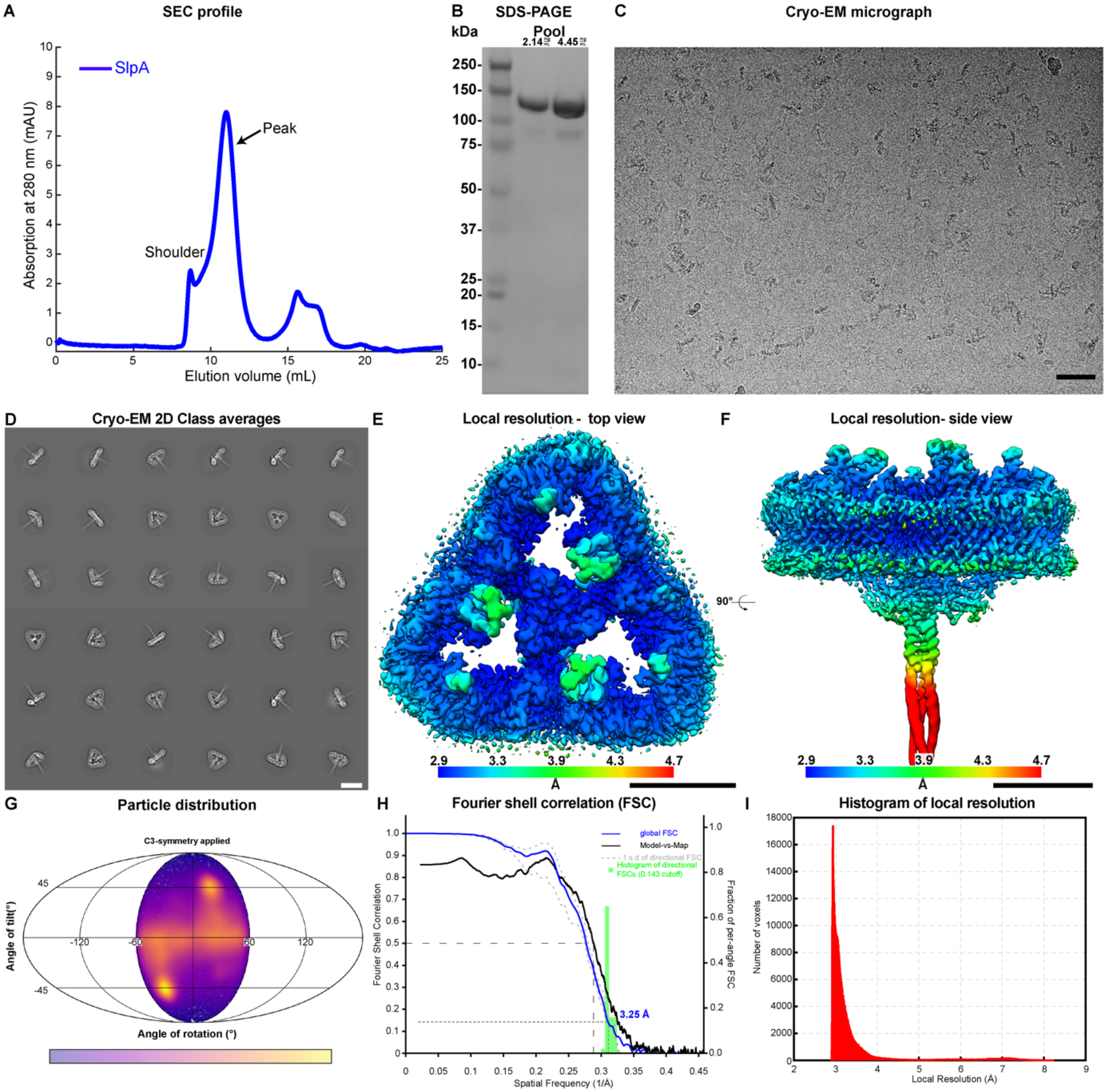

Figure S1. Cryo-EM structure of the SlpA porin of *D. radiodurans*

(A) Analytical size-exclusion chromatography (SEC) profile and (B) SDS-PAGE analysis of the final purified SlpA protein from the native source. (C) Cryo-EM image of purified SlpA protein flash frozen in liquid ethane (density black). (D) 2D Class averages of SlpA particles picked using TOPAZ (1) and classified inside RELION-3.1 (2) while ignoring CTF-correction until the first peak (density white). (E-F) Local resolution estimated in RELION, plotted into the map, shown in two orthogonal orientations. Resolution of the SlpA OMBB

core is 2.9 Å while the coiled-coil stalk is less well resolved. (G) Angular distribution of the particles in the data set, shown on a relative scale (purple denotes low and yellow denotes high). (H) 3D Fourier shell correlation between two random halves of the data (FSC) (3) and Model vs Map FSC. Histogram binning size is set to 5. (I) Histogram of local resolutions in voxels of the cryo-EM map. Scale bars: C) 500 Å; D) 200 Å; E-F) 50 Å.

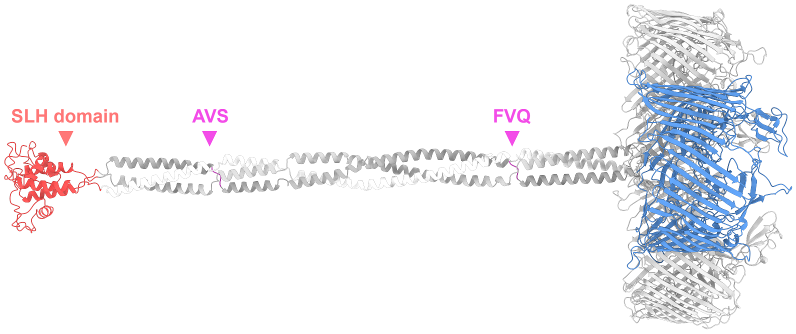

SlpA - *Deinococcus radiodurans* (UniProtKB ID: Q9RRB6)

- Signal peptide
- SLH domain; **conserved residues involved in SCWP binding are in boldface**
- Coiled-coil segment; **'a' and 'd' positions of the heptad repeats are highlighted**; **β-layer**
- Outer-membrane β-barrel; **insertions are shown in boldface**
- **Additional β-hairpin contained by comparison to orthologs with a 28-stranded β-barrel**
- Residues involved in metal-ion binding
- β-signal motif

MKKSLIALTTALSFGLAAAQTAAPVSAP
QVPALTDVPAGH**W**AKDAIDRLVSR**GVILG**YPDGTFRGTQNL**TRYE**AAIIIARLLDQMRDGETPAGMTAED
MTALQNA
IQELAAD
LAALGVR
VSDLEAN**AVS**KDD
FARLEAR
IEEVAAAGGEQGA
TEALQGQ
IDDLTAR
VDEYDALRAD
VDDNASS
IAALNDL
TVLLNQD
ILDLQDR
VSAVEAA
QAD**FVQ**RSD
FDALGGR
VTTVETR
VETVNNSLTGR
IAALERN
AFSVKPSLTIGYSVSRTSR**NFDVDRLFPLNADGTVANNAFTSGGIDTDTGAQRRDFGDFGNASDPVVAGA**
AGLYGFADGVSYTVYFTDGSTATFDGLNPADYKVPTGKVIDTTKGRNGFGFNNLARYKEGSTDIGISLGF
DTSGQFSQVTSGTGGSLFSTAGRLQVNQIDLNFGLV**TGLPSDAYVDTNGNGKKDDGEATGRGTYLGSGGT**
AAILRDPAGNVYRPVFFRFKNATTQFSVGNNPVIVTLGQQQKFYFSDYVFDNNYDGRGDGFTVTVDGSNV
PVIGAWKPQIKGVYGSRSGLDGTAEAGYGVYYRGVRAQITPVGTLTAGIHYAQEGRDMFGAAQNTTSTPS
DVTTYGADLHGKAFGVELHSEYATSRVRPNTANAAVQTSNAFYARVATRKDNLAFDLNTPAAKFGNDTFG
VSLYDLNYRKIDA**GYNNVAGISEYGYGSYSRTSAQNIAYNPDTGVTAPFANLDRQAYTDANNDGTSDRNA**
DGTVVATNTKIGQMGFGVKAAANLGPVAIGGYYDTSTGANGDNANR**MTEAGGSAKVAYSIFSLRGTYNTL**
DSNRPQIYRDAAGTQIIGDAKVRRYAVQADVTPGLGLFVGAYYRDVNV**NGVRSTTDRGLLGRGYLASSFE**
PGVGNNAYRTGLRCADNNFGTGTRDIDGVGGVLNPAVNLDQSRTATCFTSYGVEAGHAGDNANALVKDLF
FRVGYSRVYVPTTATATTGDFSGSVTYGDARYDRKVGVANVRLAGSFSTTNTQLDSRPAGTRGAVGLIVR
TDPLENVPFRPQFNGQVGYYTADNRVAAGNYNANATKYGAGVVLNDFLLPQTKIGVRYDGYMAQNRQYTP
FDGDGTQGYFSDANNNRRTNLNGVYVEGAYQDLIFSYGTYTLSQKDLNGVEYGSGINNGQPARGQTF**KIS**
YKVNF

Figure S2. Sequence annotation of the *D. radiodurans* SlpA protein.

A full structural model of *D. radiodurans* SlpA obtained by combining the cryo-EM and AlphaFold-Multimer v2.2.0 prediction is shown in cartoon representation; the two β-layers are colored in magenta (upper). The boundaries of the SLH domain, the coiled-coil segment, and the OMBB as well all other sequence features, as detailed in the rectangular box, are marked in the sequence of SlpA (lower).

```
                                   β1
D. radiodurans     (30)  AFSVKPSLTIGYSVSRTS----RNFDVDRLFPLNADGTVANNAFTSGGI------DTDTGA--QRRDFGDFGNASDPVVAGAAGLYGFADGVSYTVYFTD
D. wulumuqiensis   (30)  AFSVKPSLTIGYSVSRTN----RNFDVDRLFPLNADGTVANNAFTSGGI------DSDTGA--QRRDFGDFGNASDAVVAGEAGLYGFADGVSYTVYFTD
D. wulumuqiensis   (30)  AFSVKPSLTIGYSVSRTN----RNFDVDRLFPLNADGTVAANAFTSGGI------DADSGR--QRRDLGDFGNDSSAVVAGEGGLYGFAAAPG-------
O. desulfurans     (30)  QFTVSGSISGEYSVFRVLDGGLEDFDADRLFAT---------SFSTGEA------TTDKGDVAAKEDLGPGF----------------------------
M. ruber           (28)  RPTLTGSLSATYGYSTN-TG--GDFDLDRLFPGN--------ALSSGTGTDDVSGNRIRNA-FRRGDF--------------------------------
T. thermophilus    (28)  RYSISGSLSATYGTVVTDTG--TNFDIDRLFPGN--------AFSTGTY------GSFSSS-VQAGDS--------------------------------
```

```
                                                                            β2                              β3
D. radiodurans     (30)  GSTATFDGLNPADYKVPTGKVIDTTKGRNGFGFNNLARYKEGSTDIGISLGFDTSGQFSQVTSGTGGSLFSTAGRLQVNQIDLNFGLVTGLPSDAYVDTN
D. wulumuqiensis   (30)  GTTATYNGLNTAGYTAPDGKTVDRTKGTNGFAFNNLARYKEGSTDIGISLGFDTSGQFSQVTSGTGGSLFSTAGRLQVNQIDLNFGLVTGLPSDAYIDTN
D. wulumuqiensis   (30)  ------------------------------------AKYQEGSTNIGISLGFDTSGQFSQVTSGTGGSLFSTAGRLQVNQIDLNFGLVTGLPSDAYIDTN
O. desulfurans     (30)  -------------------------------------QQEGTWSSSLTLKLLFPNAYK-----------VDAAGLTIKGIEIDGTWDVSGGSYTGT---
M. ruber           (28)  ----------------------------------NQT-YTYGGASLNFGLKPT--------------------GGAISEVSLGLSADLVN---------
T. thermophilus    (28)  ----------------------------------NQGNISGGSASLTFGVKVAQPGTSG----------------VNVSEASATLQVPAAFGTA------
```

```
                                                                   β4                β5                              β6
D. radiodurans     (30)  GNGKKDDGEATGRGTYLGSGGTAAILRDPAGNVYRPVFFRFKNATTQFSVGNNPVIVTLGQ-QQKFYFSDYVFDNNYDGR----GDGFTVTVDGSNVPVI
D. wulumuqiensis   (30)  GNGKKDDGEATGRGTYLGSGGTAATLRDPAGNIYRPVFFRFKNATTQFSVGNNPVIVTLGQ-QQKFYFSDYVFDNNYDGR----GDGFTVTVDGSNVPVI
D. wulumuqiensis   (30)  GNGEQDAGEATGRGTYLGSGGTAATLRDSAGNIYRPVFFRFKNATTQFSVGNNPVIVTLGQ-QQKFYFSDYVFDNNYDGR----GDGFTVTVDGSNVPVI
O. desulfurans     (30)  ----------------------------------TGGFWDVSGVRTSFTVGNDPLMITLAR-QPKAHFTEYVFDNDYYSR----GTGYVVEYKGI-----
M. ruber           (28)  --------------------------------SGVARTLALNSASVKGSLSGQAFSVTYNNDDSTFSFNPYLFNNSTVGD-LITTRGFVATLDAKAFPL-
T. thermophiles    (28)  --------------------------------YTSAPTIRLNAASVKGNVDGQAFSVVYSRAVSSFKFNDYLFANDNDSEPANPRQGMVATFSATKFPL-
```

```
                                    β7                       β8              β9                        β10              β11
D. radiodurans     (30)  GAWKPQIKGVYGSRSGLDG-TAEAGYG---VYYRGVRAQITPVGTLTAGIHYAQEGRDMFGAAQNTTSTPSDVTTYGADLHGK-AFGVELHSEYATSRVR
D. wulumuqiensis   (30)  GAWKPQIKGVYGSRGGLDG-TGEAGYG---VYYRGVRAQITPVGTLTAGIHYAQEGRDLFGAAQNTTSTPSDVTTYGADLHGK-AFGVELHSEYATSRVR
D. wulumuqiensis   (30)  GAWKPQIKGVYGSRGGLDG-TGEAGYG---VYYRGVRAQITPVGTLTAGIHYAQEGRDLFGAAQNTTSTPSDVTTYGADLHGK-AFGVELHSEYATSRVR
O. desulfurans     (30)  ----LDITGVYGSTG---DADTVNGDN---RYYRGVMAGKSFEN-FSIAAYAVQEAADVYGP-------AYSNTVYGGHATAS-FGPLSLEGEYDMSNNG
M. ruber           (28)  ---SPKFTVVAGEGIDDTAIPAASRVWGTDPTYGGLRAELNLLG-ITTGLSYAEN--------------RGNRSAFGVDYKGSLFGLVNLEGAFVASTPF
T. thermophilus    (28)  ---APEVTVVAGVAGPDATKDTAPALN---GNYFGIRTAVKPFSALNLALNYATN--------------LGNRSAIGVDGGLE-LGPAKLSGLWVSSQTP
```

```
                                          β12                              β13
D. radiodurans     (30)  PNTAN---AAVQTSNAFYARVATRKDNLAFDLNTPAAKFGNDT-FGVSLYDLNYRKIDAGYNNVAGISEYGYGSYSRTSAQNIAYNPDTGVTAPFANLDR
D. wulumuqiensis   (30)  PNTAN---ATVQTSNAFYARVATRKDNLAFDLNTPAAKIGNDT-FGVALYDLNYRKIDAGYTNVAGISEYGYGSYSRTSAQNIAYNPDTGVTAPFANLDR
D. wulumuqiensis   (30)  PNTAN---AAVQTSNAFYARVATRKDNLAFDLNTPAAKIGNDT-FGVALYDLNYRKIDAGYNNVAGISEYGYGSYSRTSAQNIAYNPDTGVTAPFANLDR
O. desulfurans     (30)  T-----------PASVMYFKGGLNL------------------GDGKFKANANYRAIDPDFAGVSQD------------------------------VDT
M. ruber           (28)  GTPV-DFSNQLVTDQAFYTKLGVKL--------------------GIIELNANYRAIDPQYNNGQAGLSSASSYYYFGLGGNGEAPYGA-----------
T. thermophilus    (28)  GSPFADFFDNTLSDWAYYAQAEAKL--------------------GPLSLSANYHAVDPQYADGQAGMSENEDTTYYG-GEKAGAPYGA-----------
```

```
                                                                 β14             β15                  β16            β17
D. radiodurans     (30)  QAYTDANND----GTSDRNADGTVVATNTKIGQMGFGVKAAANL---GPVAIGGYYDTSTGANGDNANRMTEAGGSAKVAYSIFSLRGTYNTLDSNRPQI
D. wulumuqiensis   (30)  QSYTDANND----GTPDRNADGTAVVTNTKIGQMGFGVKAAANL---GPVAIGGYYDTSTGANGDNANRMTEAGGSAKLAYSIFSLRGTYNTLDSNRPQI
D. wulumuqiensis   (30)  QAYTDANND----GTSDRNADGTVVATNTKIGQMGFGVKAAANL---GPVAIGGYYDTSTGANGDNANRMTEAGASAKLAYSIFSLRGTYNTLDSNRPQI
O. desulfurans     (30)  AGYQSEDSSGNTINGAPFGD-----------DQKGYGFDAAAEL---GAFSVSGYYDHQTDFAGTATSQVDKYGAAAEAKLSVITLKGNYDYYNA-----
M. ruber           (28)  -------------------------------NNRGFGLDGKLTLPILGGIEVRGFYDNSADFATGAQ---------------------------------
T. thermophilus    (28)  -------------------------------DTRGLGVSASVGF---GPVTLKGYAESEGDYNLAPG---------------------------------
```

```
                                            β18              β19
D. radiodurans     (30)  YRDAAGTQIIGDAKVRRYAVQADVTPGLGLFVGAYYRDVNVNGVRSTTDRGLLGRGYLASSFEPGVGNNAYRTGLRCADNNFGTGTRDIDGVGGVLNPAV
D. wulumuqiensis   (30)  YRDAAGTQIIDDAQVRRYAVQADVTPGLGLFVGAYYRDVNVNGVRSTTDRGLLGRGYLASSFEPGVGNNAYRTGLRCADNNFGTGTRDIDGVGGVLNPAV
D. wulumuqiensis   (30)  YRDAAGTQIIDDAQVRRYAVQADVTPGLGLFVGAYYRDVNVNGVRSTTDRGLLGRGYLASSFEPGVGNNAYRTGLRCADNNFGTGTRDIDGVGGVLNPAV
O. desulfurans     (30)  ------DATDPTNDEVTYGGSAAIGPMKGFTLEAFYSLATLGGA------------------------------------------------------AVDG
M. ruber           (28)  -------------TADTFSVGATIPLFAGFSLNPFFNNTNVNGAQVSGATGGQSGLNGNY----------------------------------------NY
T. thermophilus    (28)  ------------SVNDAWGVAATLGSFRGFSLTGFYNAAYTGGNGYFSLTTAVDAIAPG-----------------------------------------VT
```

```
                                          β20                β21                       β22            β23
D. radiodurans     (30)  NLDQSRTATCFTSYGVEAGHAGDNANALVKDLFFRVGYSRVYVPTTATATTGDFSGSVTYGDARYDRKVGVANVRLAGSFSTTNTQLDSRP--AGTRGAV
D. wulumuqiensis   (30)  NLDQSRTSTCFTSYGVEAGHAGDNANALVKDLFFRVGYSRVYVPTTATATTGDFSGSVAYGDARYDRKVGVANVRLAGSFSTTNTQLDSRP--AGTRGAV
D. wulumuqiensis   (30)  NLDQSRTSTCFTSYGVEAGHAGDNANALVKDLFFRVGYSRVYVPTTATATTGDFSGSVAYGDARYDRKVGVANVRLAGSFSTTNTQLDSRP--AGTRGAV
O. desulfurans     (30)  PLPT---TEGESAYGASLSHDGASENALISGLNLTAKYKAYTVS--------GNTDIQVYGD--FDAKFGVVGIHPMFRYHMPNP--------GDTTIKY
M. ruber           (28)  YLYNNQDARWSSGFGVKLIHNGRASNALIPNLDINLWYQSFAGPN-------AGNDVLASVG--YNAKLGFISFNPILRYHSFSAAAPAPGNRSTNALKY
T. thermophilus    (28)  YYYTIENQKYSSSWGVRVAHDGKAEDALIPTLNLTAQYATYYVS--------GHTDIQVYADLAKPFKLAILSLSPGFRYHSFAGAGSAP---TYTTLKG
```

```
                           β24                 β25                    β26                  β27
D. radiodurans     (30)  GLIVRTDPLENVPFRPQFNGQVGYYTADNRVAAGN--YNANATKYGAGVVLNDFLLPQTKIGVRYDGYMAQNRQYT------PFDGDGTQGYFSD-----
D. wulumuqiensis   (30)  GLIVRTDPLANLPFRPQLNGQVAYYTADNRLGTGNLTYNANATKYGAGVVLNDFLLPQTKIGVRYDGYVAQNRQYT------PFDGSGTQGFFSD-----
D. wulumuqiensis   (30)  GLIVRTDPLANLPFRPQLNGQVAYYTADNRLGTGNLTYNANATKYGAGVVLNDFLLPQTKIGVRYDGYVAQNRQYT------PFNGSGTQGFFSD-----
O. desulfurans     (30)  GAQVKVGEL-GVVFKPTLMGDFVSRTTG----------TTSEMKYGVGLELGDFVFG-SSLKGGYASYTAANVASV-----LLADAQLLDPFNPADDRVW
M. ruber           (28)  GIGVTTDQL-GIFLKPSLEGAFVQQSITETAPGSN---SSSEQYWRVGLNLNEFLATGSVFKVGYARYSATNVVSPNPVGGAPARGFGNFPLSLTTDRVF
T. thermophilus    (28)  GVQVSTDPL---LFGLSLDGAVSYRRTQYTNNPSN--VTTYELYYRAGVKLQDFLAPKLNFSVAYAHYEGDQLAGT----GLPVVGSGNQAFNFARDRVY
```

```
                                                  β28              β29                            β30
D. radiodurans     (30)  --------------ANNNRRTNLNGVYVEGAYQDLIFSYGTYTLSQKDL-NGVEYGSGINNGQPARGQTFKISYKVNF
D. wulumuqiensis   (30)  --------------SNNNRRTNLNGVYVEGAYQDLIFSYGTYTLSQKDMTSGTEYGSGIGNGQPARGQTFKISYKVNF
D. wulumuqiensis   (30)  --------------SNNNRRTNLNGVYVEGAYQDLIFSYGTYTLSQKDM-AGTEYGSGLNDGKAARGQTFKISYKVNF
O. desulfurans     (30)  SS-------------TGTTNGSLTGYFFEWTYEGMQFAYIDAVVNN--------------GGNTTHGQAFKVSYSVEF
M. ruber           (28)  NYPGEVQFPWNLTTDFGTNSGSVTGLYLEWKYGNLTMAAASATLADG------------SGATVSNGSGFKISYEVKF
T. thermophilus    (28)  RSPDPIAAPWLA--TPGTQAGKLDGFYIEAKYYDLTVAYGEFVLDDL------------NGTNPNFGRGFKISYTVKF
```

Figure S3. Multiple sequence alignment of the OMBB region of representative SlpA proteins.

Residues part of β-strands are underlined in the sequence of *D. radiodurans*. Large insertions in *D. radiodurans* OMBB are shown in boldface, the extra β-hairpin is colored red, and the β-signal motif (BAM insertion signal) is colored green. The number of β-strands contained in the individual OMBBs are indicated with rounded brackets. Accession details for the shown sequences are provided in Table S2.

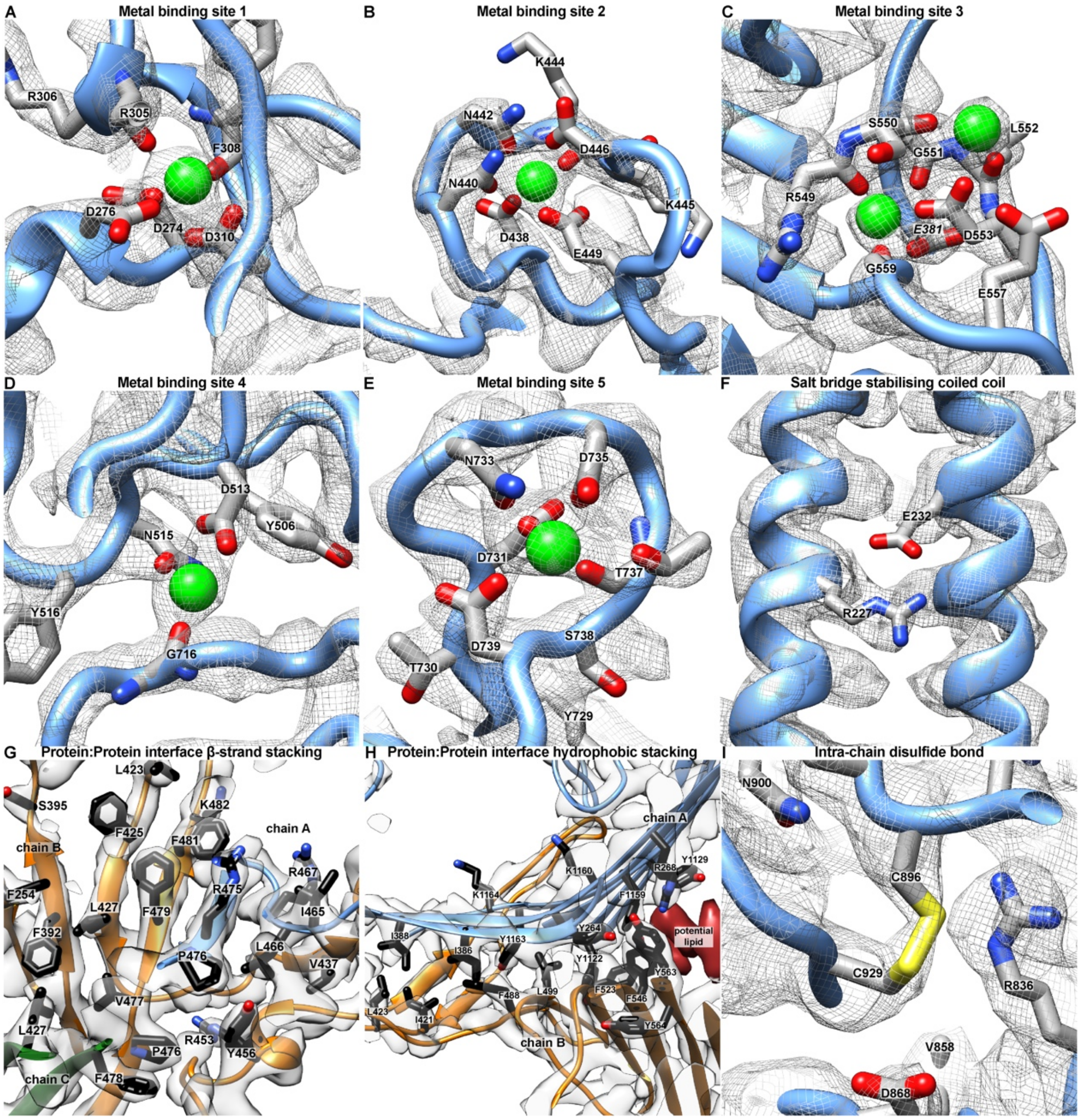

Figure S4. Close-up views of the SlpA OMBB model.

(A-E) Close up views of putative metal ion (green) binding sites in SlpA, depicted as ribbon and stick diagram. In all metal binding sites, putative metal ions are coordinated by carboxyl group of aspartate and glutamate residues as well as the carbonyl oxygen of asparagine side chains or the main chain peptide bond. While there is clear density for the metal ion, the chemical identity of the metal cannot be ascertained. (F) A prominent salt bridge between the E232 and R227 of a second SlpA subunit stabilizes the coiled-coil region. (G) Close to the central symmetry axis the trimeric conformation is stabilized by

hydrogen bonding via β-strand stacking. (H) The β-sheets of two opposing outer membrane barrels are stabilized through hydrophobic stacking (see also Figure 2C,D). Additional density at the interface could correspond to lipid molecules, however we cannot assign this unambiguously (marked in mud-colored density). (I) The primary sequence of SlpA contains two cysteine (896, 929) residues which form an intra-chain disulfide bond within the plug of the OMBB (resolved). In panels A-F) and I) cryo-EM density is shown as grey mesh, while in panels G-H), density is shown as an isosurface for clarity.

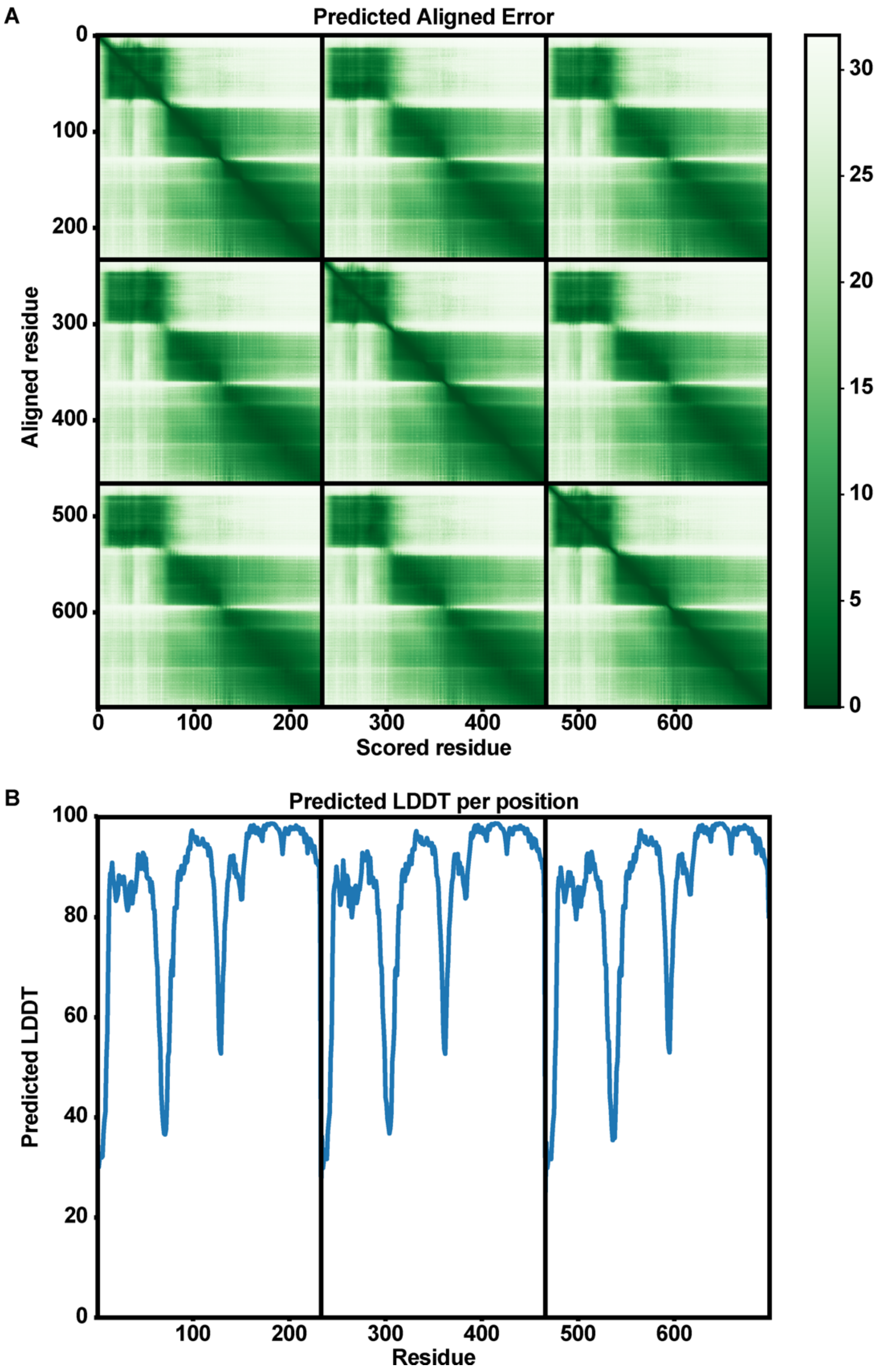

Figure S5. AlphaFold modelling of the SlpA periplasmic segment. The homotrimeric periplasmic part of SlpA was modelled using AlphaFold-Multimer v2.2.0. The Predicted Aligned Error (PAE, upper) and per-residue confidence (pLDDT, lower)

plots for the model with the best confidence is shown. Low PAE and high pLDDT values are indicators for high accuracy of the model yielded by AlphaFold-Multimer v2.2.0. The subplots in each panel correspond to one SlpA monomer.

```
D. radiodurans      [99] MTALQNAIQELAADLAALGVRVSDLEANAVSKDDFARLEARIEEVAAA-----GGEQGATEALQGQIDDL
D. wulumuqiensis    [99] MTALQNAIQELAADLAALGVRVSDLEANAVSKDDFARLEARIEEVAAA-----GGEQGASEDIQGQIDEL
D. wulumuqiensis    [99] MTALQNAIQELAADLAALGVRVSDLEANAVSKDDFARLEARIEEVAAA-----GGEQGASEDIQGQIDEL
D. ficus           [101] LTALQNAVQELAADLAALGVRVTDLEENMVSKEDFARLEERVNALGAV-----EGDPTALQGITDQLAAL
D. chartae         [102] VTTLRNAVQELAADLAALGVRVADLEDNAVTKDDFARLEEQVNTLAGAT----GSDPEALKELTDQLEAA
M. hydrothermalis   [90] LTALRNAVQELAAELSSLGVRVGALEDNAATKDDVARLEAAINELRAQPVPEPGVDEKALRELAERVEAA
O. desulfurans      [89] ITALRNAVQELAAELASLGVRVSALEDNAASKSDVARLEKMIAELKGMPSGE-GASGAALKDLADRVEAA
M. ruber            [96] MTAIRNAVQELAAELAALGVRVSALEDNAASKDDIARLEAAIEALKAAPAPAPGMDEAALADLADRVEAA
T. thermophilus     [98] LEALKNAVQELAAELASLGVRVSALEDSAATKEDIARLEAMIAELKAQPMPEPGMDQAALKDLMDRVEAA
T. thermophilus     [98] -------------------------------------LEAMIAELKAQPMPEPGMDQAALKDLMDRVEAA


D. radiodurans     [164] TARVD-------------------------EYDALRADVDDNASSIAALNDLTVLLNQDILDLQDRVSAV
D. wulumuqiensis   [164] TARVD-------------------------EYDALRADVDDNASSIAALNDLTVLLNQDILDLQDRVSAV
D. wulumuqiensis   [164] TARVD-------------------------EYDALRADVDDNASSIAALNDLTVLLNQDILDLQDRVSAV
D. ficus           [166] NTSVDELTA---------------------NYDTLRADVDDNASNIAALNDLTVLLNQDILDLQDRVSAV
D. chartae         [168] SIAADTALAQATELQDK-------FEALDGRVSELAAEVEANAASIAALNDLTVLLNQDILSLQDRVTAL
M. hydrothermalis  [160] SIAADTALAQAQQLNEQ-------LGAVEGDLAALRSLVEANADSIKALNDLAVLLNQDVLELQDRVTAL
O. desulfurans     [158] AIAADTALAQ--------------VQALEGKVDAVGAQASANADSIKALNELAVLLNQDVLSLQDRVTAL
M. ruber           [166] SVAADTALAQAQVLAER-------LDGIEGDVAALKTQVEADADSIRALNELAVLLNQDVLSLQDRVTAL
T. thermophilus    [168] SIAADTALAQAQQLAERLDALAQDVEGVKGDLAGLRSQVEANADAIQALNELAVLLNQDVLSLQDRVTAL
T. thermophilus    [131] SIAADTALAQAQQLAERLDALAQDVEGVKGDLAGLRSQVEANADAIQALNELAVLLNQDVLSLQDRVTAL


D. radiodurans     [209] EAA-----------------------QADFVQRSDFDALGGRVTTVETRVETVNNSLTGRIAALERN
D. wulumuqiensis   [209] EAA-----------------------QADFVQRSDFDALGGRVTTVETRVETVNNTLSGRIAALERN
D. wulumuqiensis   [209] EAA-----------------------QADFVQRSDFDALGGRVTTVETRVETVNNTLSGRIAALERN
D. ficus           [215] EAA-----------------------QSDFVLRADFDNLTNRVAGIDTRVTNLEKA-----------
D. chartae         [231] ETE------------GVTPDDLEALREFSTLTRRDLTALTDRVEGIDTRVAALESA-----------
M. hydrothermalis  [223] EKVGQ-----PDVSGLASQDQVVAVQEFATALRNDLVNLSNRVSALDTQVADIDER----LQTVEAN
O. desulfurans     [214] EKASGM----TDLSGVATKDDVQSVRDYVTAIRGDLVNVSNKVSALEANVGDLQDQ----VNGLKFY
M. ruber           [229] EKQLGD----VDFESFANREDVSAIQEFATALRSDLVRLSDRVSALDTRVGALDQR----LAAVEAT
T. thermophilus    [238] EKMVSGGQELPDLEQFATKEDVAAVQEFAAALRSDLVGLSDKVSKLEEQVAELNKV-----------
T. thermophilus    [201] EKMVSGGQELPDLEQFATKEDVAAVQEFAAALRSDLVGLSEKVSKLEGTVGDLSGK----VATLQRN
```

Figure S6. Multiple sequence alignment of the coiled-coil region of representative SlpA proteins.

The core-forming hydrophobic positions (‘a’ and ‘d’ positions of the heptad repeats in canonical coiled coils) are shown in boldface in each sequence. Residues involved in forming β-layers are coloured red. Accession details for the shown sequences are provided in Table S2.

A

Multiple sequence alignment of SLH domains from SlpA-like proteins

**SlpA**		
Deinococcus radiodurans	[32]	ALTDVPAGH**W**AKDAIDRLVSR-**G**VIL**GY**PDGTFRGTQNL**TRYE**AAIIIARLLDQMRDGET
Deinococcus wulumuqiensis	[32]	ALTDVPAGH**W**AKDAIDRLVSR-**G**IIL**GY**PDGTFRGTQNL**TRYE**AAIIIARLLDQMRDGET
Deinococcus wulumuqiensis	[32]	ALTDVPAGH**W**AKDAIDRLVSR-**G**IIL**GY**PDGTFRGTQNL**TRYE**AAIIIARLLDQMRDGET
Deinococcus ficus	[33]	ALTDVPAGH**W**AKDAIDKLVSR-**G**IIL**GY**PDGTFRGTQNL**TRYE**AAVIIARLLDQMRSGEV
Deinobacterium chartae	[37]	ALSDVPAGH**W**AKDAVDQLVAK-**G**IIT**GF**PDGTFRGNEGL**TRYQ**AALIIARVLEQVAAGSV
Marinithermus hydrothermalis	[24]	QFSDVPAGH**W**AKEAVEKLADE-**G**IIL**GF**PDGTFRGNEGL**TRYQ**AALIIFRVLETIREEQL
Oceanithermus desulfurans	[23]	QFSDVPAGH**W**AKEAVEKIAAE-**G**IIL**GF**PDGTFRGNENL**TRYQ**AAMIIYRLLQKLEPGQM
Meiothermus ruber	[24]	QFSDVPAGH**W**AKEAVERIAAC-**G**LIT**GF**PDGTFRGNTNL**TRYQ**AALIFQRLLNEIQQGGE
Thermus thermophilus	[24]	QFSDVPAGH**W**AKEAVEALAAK-**G**IIL**GF**PDGTFRGNENL**TRYQ**AALLIYRLLQQIEEELK
Thermus thermophilus	[24]	QFSDVPAGH**W**AKEAVEALAAK-**G**IIL**GF**PDGTFRGNENL**TRYQ**AALLIYRLLQQIEEELK
**SlpA-like**		
Deinococcus radiodurans	[36]	QFTDVPAGH**W**AKDAVDRITQC-**G**LIQ**GF**PDGTFRGNENL**TRYQ**AALIFYRMLSTNALSTC
Deinococcus radiodurans	[45]	APVACTQGA**W**AKAAIDLVTQK-**G**LFI**GY**PDGSFDWCSAI**TRQE**VAQVLARLLAQMPENTF
Thermus thermophilus	[23]	--QDAPPSP**W**AEEAVRILVAK-**G**VFI**GY**PDGSFRWREPM**TRQE**AAALALYRLLAAYGLDRL
Armatimonas rosea	[24]	PFKDVPNDH**W**AYQAIDKLAQL-KIII**G**DPDGQFHGKRTL**TRYE**MAVMLARLLQQIEEELK
Armatimonas rosea	[26]	APDDVPATH**W**AYPAVQDLISK-**G**LIQN**F**PNGKYLGERTL**TRYE**MASLVKRMLDYLAKTQV
Acetomicrobium mobile	[25]	PFVDVPMNH**W**AYDAISQLAAK-**G**IIQ**GY**PDGTYRGNHPM**TRYE**MSMLVARALATVDMEKA
Acetomicrobium mobile	[25]	PFVDVPMNH**W**AYDAISQLAAK-**G**IIQ**GY**PDGTYRGNQPM**TRYE**MSMLVARALATVDMEKA
Synechococcus sp. PCC 7502	[74]	QLSDVQPTD**W**AFTSLQSLVERY**G**CIA**GY**PDKTYRGQRAL**SRYE**FAAGLNACLDKVNELIS
Synechococcus sp. PCC 7502	[64]	QFSDVQPTD**W**AFTALQSLVERY**G**CIA**GY**PDSTYRGSRAL**SRYE**FAAGLNACLDKINELIS
Synechococcus sp. PCC 7502	[55]	QLSDVQPTD**W**AFTALQSLVERY**G**CIA**GY**PNGTYRGSRAL**SRYE**FAAGLNACLDKINELIS
Synechococcus sp. PCC 7502	[74]	QLSDVQPTD**W**AFTALQSLVERY**G**CIA**GY**PNGTFRGSRAT**SRYE**FAAGLNACLDKINELIS
Synechococcus sp. PCC 7502	[56]	QLSDVQPTD**W**AFTSLQSLVERY**G**CIA**GY**PNATFRGNRVL**SRYE**FAAGLNACLDKINQIIS
Synechococcus sp. PCC 7502	[50]	QLLDVQPTD**W**VFVAVQSLVERYSCLE**GY**PSHKYFGTRVL**SRYE**FASGLNACINHINQLLT
Ca. *Melainabacteria* bacterium	[31]	NFADMPAEH**W**AAKAVTDLVEKY**G**VMA**GF**PDQTFKGTRNI**SRYE**AAAAFYKVMLQMSQVED
Ca. *Saganbacteria bacterium*	[23]	KFKDLPADH**W**AAKSVYSLVKM-**G**VTS**GY**PDGTFRGKKNI**TRYE**TAIFLSKLADRLKDEVA
Chloroflexaceae bacterium	[67]	QLRDVNPGE**W**AFEALRSLVERY**G**CIE**GY**PDQTFRGNRAT**TRYE**FAAGLNSCLNSIERLIA
Chloroflexaceae bacterium	[32]	QLRDVSPGE**W**AFEALRSLVERY**G**CIE**GY**PDQTFRGNRAL**TRYE**FAAGLNACLNSVERLIA
Chloroflexaceae bacterium	[81]	ELQDVSPSD**W**AFEALRSLVERY**G**CIE**GY**PDRTYRGNRAL**TRYE**FAAGLNSCLNAIERLIA
Chloroflexaceae bacterium	[34]	QLRDVQPSD**W**AYQALQLLIERYNCLT**GY**PDASFRGHRAL**TRYE**FAAGLNACLDQVEGLVR
Acetohalobium arabaticum	[25]	PFTDVPADH**W**AYDAIKEVSEA-**G**IVT**GY**EDGTFKGDEKL**TRYE**MAVIAARISSQVEEGEA
Halanaerobium saccharolyticum	[24]	SFSDVPSDH**W**AYDAINKLVAA-**G**IVE**GY**PDGEFKGQQSM**TRYE**MAVMVSRALDKIADEQQ
Acidaminococcus fermentans	[24]	PFSDLPAGH**W**AYGAVAKLAAA-**G**VVD**GY**PDGTFKGDKTM**TRYE**MAQIVAKALAKGAIGAD
Acidaminococcus fermentans	[24]	PFSDLPAGH**W**AYGAVAKLAAA-**G**VVD**GY**PDGTFKGDKTM**TRYE**MAQIVAKALAKGAIGAD
Acidaminococcus fermentans	[24]	PFSDLPAGH**W**AYGAVAKLAAA-**G**VVD**GY**PDGTFKGDKTM**TRYE**MAQIVAKALAKGAIGAD
Acidaminococcus fermentans	[24]	PFSDLPAGH**W**AYGAVAKLAAA-**G**VVD**GY**PDGTFKGDRTM**TRYE**MAQIVAKALAKGAIGAD
Acidaminococcus fermentans	[24]	PFSDLPAGH**W**AYGAVAKLAAA-**G**VVD**GY**PDGTFKGDKTM**TRYE**MAQIVAKALAKGAIGAD
Limnochorda pilosa	[28]	PFPDVPPDH**W**AAESVELLRAA-**G**LVI**GY**PDGEYKGNRQL**TRYE**WAMIVSRLVDRLDAMVA
Cytophagales bacterium	[31]	PFADVPTDH**W**AYQSVDKLQKG-**G**IVI**GY**PDGTYGGKRAM**TRYE**FAVAIARLLEKIPQPDL
Parabacteroides distasonis	[25]	PFSDVTPDS**W**AYQAVSQLAST-**G**IIT**GY**PDGTFRGENGI**TRFQ**VAQMVAKALANQDRANA
Salmonella enterica subsp.	[25]	PFSDVEPSS**W**AYQSVEQLASA-**G**IIN**GY**PDGTFKGNKDI**TRYE**MAQMVAKAMANQDRANA
Salmonella enterica subsp.	[25]	PFSDVTPDS**W**AYQAVSQLAST-**G**IIT**GY**PDGTFRGENGI**TRFQ**VAQMVAKALANQDRANA
**Ompα**		
Thermotoga maritima (Ompα/OmpA1)	[21]	FFPDVPKDH**W**AYEYVWKLWQR-**G**IFI**GY**PDGEFKGDRYI**TRYE**AATAVSRLLDFIEQKML
Thermotoga maritima (OmpA2)	[20]	QFKDVPVNH**W**AYEAVMEMSKL-**G**VLT**G**MPDGTFQGNSYL**TRYQ**AAVAFYRLYNILKQPSA
Thermotoga maritima (OmpA3)	[23]	NIKDLEPDSPFFEAVNYVVKA-**G**IMELDDKGNFRGALLV**TRYD**VAQYIYRLVMRFELEKL
Fervidobacterium nodosum (Ompα)	[20]	AFRDIPKGH**W**AKNYVERLEQI-**G**IVT**GF**PDGTYRGDEAV**TRYQ**IALFISRTLDYVEQSLS

B

Structure comparison of the SCWP-binding sites of SpaA and SlpA

***Paenibacillus alvei* SpaA**
SLH domain pseudotrimer (PDB 6CWI)

***Deinococcus radiodurans* SlpA**
SLH domain homotrimer (AlphaFold2 model)

Superposition of SpaA and SlpA
SLH domain trimers

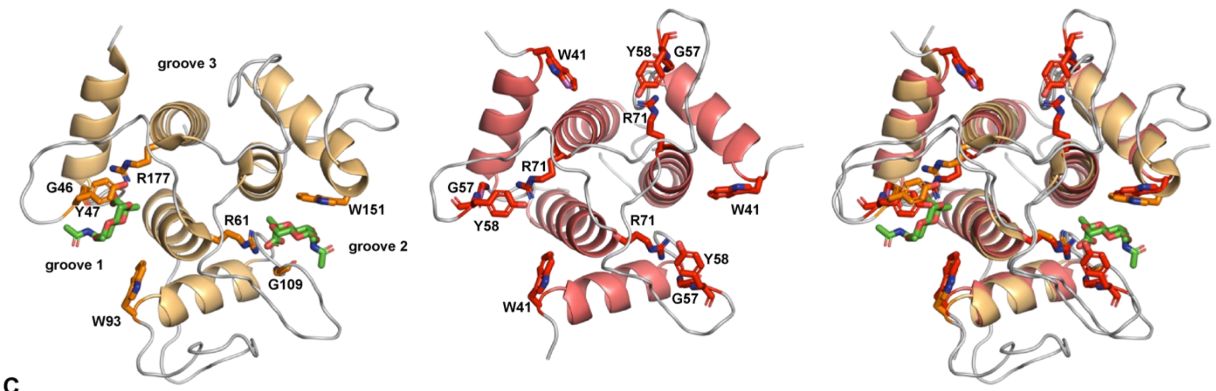

C

		hhhhhhhhhhhh *hhhhhhhhhhhhhhhh*	
D. radiodurans SlpA	[39]	GH**W**AKDAIDRLVSR**G**VIL**GY**--PDGT-FRGTQNL**TR**Y**E**AAIIIARLLDQMR	[86]
P. alvei SpaA	[28]	AKTTQEKFDALKEA**G**VFS**GY**--PGTTDAKLGQDM**TRAE**FAKVLVKLFGLKE	[76]
P. alvei SpaA	[91]	KN**W**AAPFIEAVTAE**G**LMQ**G**KDLTKKI-FDFNGKI**T**VE**E**ASKTLVTALKLEP	[140]
P. alvei SpaA	[149]	TD**W**AKGYFEAAVNA**G**LFSKD-------ANPKANA**TRAQ**LVEAAFAADEMSK	[192]

Figure S7. Sequence and structural comparisons of SLH domains from SlpA and SlpA-like proteins.

(A) Residues important for binding PG-linked SCWPs are shown in boldface, and the start position of the SLH domain is indicated in brackets. Accession details for the shown sequences are provided in Table S2. (B) The S-layer protein SpaA of *Paenibacillus alvei* CCM 2015^T contains three consecutive copies of the SLH domain in its N-terminal segment (4). These three domains form a pseudotrimer, similar to the homotrimer formed by the SLH domain of *D. radiodurans* SlpA. Side chains of the residues involved in SCWP-binding in grooves 1 and 2 of SpaA and the corresponding residues of SlpA are shown in stick representation. (C) A sequence alignment of the SLH domains of SpaA and SlpA is additionally shown. Residues involved in SCWP-binding in SpaA are colored red. The TRAE and GhhxG (h=hyphobhic amino acid residue) motifs, which are highly conserved in SLH domains, are shown in boldface. Secondary structure (*h*=α-helix) observed in the SLH domain of SlpA is marked.

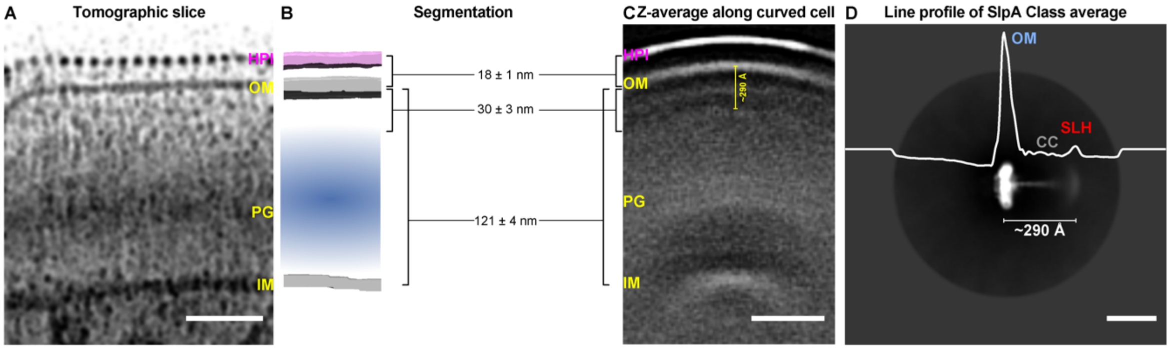

Figure S8. Cryo-ET and cryo-EM of *D. radiodurans* cell envelopes.

(A) Tomographic slice through the outer envelope of a partially lysed *D. radiodurans* cell. The cell envelope can be divided into four prominent regions, as listed from the extracellular space to the cytosol: A repetitive S-layer containing the HPI protein, the OM, a thick layer of PG, and the IM, which is in agreement with previous studies (5). (B) Segmentation of the tomographic volume. (C) Z-average of subtomograms extracted along the curved surface of *D. radiodurans* highlights the most prominent cell surface features of the complex envelope of *D. radiodurans*. A weak PG density appears, ~290 Å away from the OM towards the interior of the cell (marked). (D) Line profile of 2D cryo-EM class average of SlpA with the same distance of the OMBB to the S-layer homology (SLH) domain (marked). Binding of the SlpA SLH domain to the *D. radiodurans* PG has been previously biochemically demonstrated (6). Scale bar: A,C: 500 Å D) 200 Å.

Supplementary Tables

Table S1: Cryo-EM data collection, refinement and validation statistics

	#Drad_SlpA (EMD-15378) (PDB ID 8AE1)
**Data collection and processing**	
Microscope	Titan Krios G3
Magnification	81,000
Voltage (kV)	300
Total Electron exposure ($e^{-}/Å^2$)	47.909
Detector	K3 (Gatan)
Slit width (eV)	20
Defocus range (µm)	-1 to -2.5
Acquisition Mode	Super-resolution
Pixel size (Å)	0.546
AFIS[&] Mode	Yes
Micrographs collected	2,294
Micrographs used	2,294
**Data processing**	
Software reconstruction	RELION3.1 (2)
Software picking	TOPAZ (1)
Initial particle images (no.)	223,878
Final particle images (no.)	122,412
Rescaled Box-size Class2D (px)	128 x 128
Rescaled Box-size Class3D (px)	512 x 512 x 512
Final Box-size (px)	512 x 512 x 512
Pixel size final reconstruction (Å)	1.092
Symmetry imposed	C3
Map resolution (Å)	3.25
FSC threshold	0.143
Map resolution range (Å)	2.9 – 6.7
Map sharpening *B* factor ($Å^2$)	-46.8126
3DFSC sphericity[#]	0.985
**Model Refinement**	
Initial model used (PDB code)	None

Software	PHENIX (7)
Model resolution (Å)	3.3
FSC threshold	0.5
Model composition	
Non-hydrogen atoms	21,504
Protein residues	2,859
Ions	18
B factors (Å^2)	
Protein	51.67
Ligand	58.42
R.m.s. deviations	
Bond lengths (Å)	0.002
Bond angles (°)	0.472
Validation	
MolProbity score	1.29
Clashscore	3.61
Poor rotamers (%)	0.00
Cβ outliers (%)	0.00
CABLAM outliers (%)	2.14
Ramachandran plot	
Favored (%)	97.27
Allowed (%)	2.73
Disallowed (%)	0.00
Rama-Z (Z-score, RSMD)	
whole (N= 2853)	-1.01 (0.16)
helix (N= 183)	-0.65 (0.42)
sheet (N= 1236)	-0.39 (0.15)
loop (N= 1434)	-0.83 (0.16)

& AFIS: Aberration Free Imaging Shift Mode.

3D-FSC sphericity as determined by the methods described in(3).

Table S2: Accession information for SlpA and SlpA-like proteins; several bacteria contain multiple homologs.

Organism	NCBI/UniProt accession	Barrel size
**SlpA**		
Deinococcus radiodurans	WP_010889202	30-stranded
Deinococcus wulumuqiensis	WP_017871655	30-stranded
Deinococcus wulumuqiensis	WP_114672325	30-stranded
Deinococcus ficus	WP_191241882	28-stranded
Deinobacterium chartae	WP_183988817	28-stranded
Marinithermus hydrothermalis	WP_013702952	30-stranded
Oceanithermus desulfurans	WP_147148761	30-stranded
Meiothermus ruber	WP_013015232	28-stranded
Thermus thermophilus	WP_011228992	28-stranded
Thermus thermophilus	P35830	28-stranded
**SlpA-like**		
Deinococcus radiodurans	WP_010887767	8-stranded
Deinococcus radiodurans	WP_027480303	8-stranded
Thermus thermophilus	WP_011228967	8-stranded
Armatimonas rosea	WP_184200374	20-stranded
Armatimonas rosea	WP_184200710	16-stranded
Acetomicrobium mobile	WP_014807739	18-stranded
Acetomicrobium mobile	WP_014806458	18-stranded
Synechococcus sp. PCC 7502	WP_015167070	16-stranded
Synechococcus sp. PCC 7502	WP_015168280	16-stranded
Synechococcus sp. PCC 7502	WP_015168863	16-stranded
Synechococcus sp. PCC 7502	WP_015168481	16-stranded
Synechococcus sp. PCC 7502	WP_041429169	16-stranded
Synechococcus sp. PCC 7502	AFY74914	16-stranded
Ca. *Melainabacteria* bacterium	PKL78650	18-stranded
Ca. *Saganbacteria bacterium*	MBI5699473	18-stranded
Chloroflexaceae bacterium	NJL83094	16-stranded
Chloroflexaceae bacterium	NJL82168	16-stranded
Chloroflexaceae bacterium	NJL82038	16-stranded
Chloroflexaceae bacterium	NJL82082	16-stranded
Acetohalobium arabaticum	WP_013279101	24-stranded
Halanaerobium saccharolyticum	WP_133516444	22-stranded
Acidaminococcus fermentans	WP_106787916	16-stranded
Acidaminococcus fermentans	WP_012939007	16-stranded
Acidaminococcus fermentans	WP_012939010	16-stranded
Acidaminococcus fermentans	WP_012939008	16-stranded
Acidaminococcus fermentans	WP_012939009	16-stranded
Limnochorda pilosa	WP_068139859	14-stranded
Cytophagales bacterium	MBC8103537	20-stranded
Parabacteroides distasonis	WP_172733356	16-stranded
Salmonella enterica subsp.	EBY4570518	16-stranded
Salmonella enterica subsp.	EBY4570338	16-stranded
**Ompα and Ompβ**		
Thermotoga maritima (Ompα/OmpA1)	Q01969	-
Thermotoga maritima (OmpA2)	Q9X252	-
Thermotoga maritima (OmpA3)	Q9WZH0	-
Thermotoga maritima (Ompβ)	WP_004081490	22-stranded
Fervidobacterium nodosum (Ompα)	ABS61559	-
Fervidobacterium nodosum (Ompβ)	ABS61560	24-stranded

Table S3. OMBB proteins in *D. radiodurans*; proteins for which we are unsure about the number of β-strands are indicated by (?).

NCBI accession	Barrel size	Remarks
Q9RRB6	30-stranded	SlpA
AAF11946	38-stranded	Contains N-terminal immunoglobulin-like (invasin D3) domains
UID71982	38-stranded	Contains N-terminal immunoglobulin-like (invasin D3) domains
UID69687	38-stranded	Contains N-terminal immunoglobulin-like (invasin D3) domains
AAF11400	8-stranded	No additional domains
ANC71147	8-stranded	Contains a C-terminal domain with a cystatitin-like (NFT2-like) fold
ANC71478	18-stranded	No additional domains
AAF10759	8-stranded	No additional domains
AAF10697	8-stranded	SlpA-type
AAF10549	8-stranded	No additional domains
UID69670	8-stranded	No additional domains
AAF10255	8-stranded	No additional domains
AAF10153	38-stranded (?)	LPS-assembly protein LptD
AAF09960	16-stranded	Outer membrane assembly factor BamA
ANC72597	8-stranded	SlpA-type
QIP33551	24-stranded	PapC usher
ANC70518	12-stranded (?)	No additional domains
AAF09880	12-stranded (?)	No additional domains
AAF11062	8-stranded	No additional domains
AAF10975	8-stranded	No additional domains

Supplementary Movie Legend

Movie S1. Cryo-EM reconstruction of SlpA from *D. radiodurans*

The 3.25 Å (global) resolution cryo-EM reconstruction is shown with the atomic model (ribbon diagram) built into the density (isosurface). Resolution of the OMBB core is 2.9 Å (see Figure S1).

Supplementary References

1. T. Bepler *et al.*, Positive-unlabeled convolutional neural networks for particle picking in cryo-electron micrographs. *Nat Methods* **16**, 1153-1160 (2019).
2. J. Zivanov, T. Nakane, S. H. W. Scheres, Estimation of high-order aberrations and anisotropic magnification from cryo-EM data sets in RELION-3.1. *IUCrJ* **7**, 253-267 (2020).
3. Y. Z. Tan *et al.*, Addressing preferred specimen orientation in single-particle cryo-EM through tilting. *Nat Methods* **14**, 793-796 (2017).
4. R. J. Blackler *et al.*, Structural basis of cell wall anchoring by SLH domains in *Paenibacillus alvei*. *Nat Commun* **9**, 3120 (2018).
5. D. L. Sexton, S. Burgold, A. Schertel, E. I. Tocheva, Super-resolution confocal cryo-CLEM with cryo-FIB milling for in situ imaging of *Deinococcus radiodurans*. *Current Research in Structural Biology* **4**, 1-9 (2022).
6. C. S. Misra, B. Basu, S. K. Apte, Surface (S)-layer proteins of *Deinococcus radiodurans* and their utility as vehicles for surface localization of functional proteins. *Biochim Biophys Acta* **1848**, 3181-3187 (2015).
7. D. Liebschner *et al.*, Macromolecular structure determination using X-rays, neutrons and electrons: recent developments in Phenix. *Acta Crystallogr D Struct Biol* **75**, 861-877 (2019).
